# Supplementary material for: Key innovations and the diversification of Hymenoptera
Source: Nat Commun. 2023 Mar 3;14:1212. doi: 10.1038/s41467-023-36868-4 (PMC9984522; doi:10.1038/s41467-023-36868-4)
Supplement: Supplementary file 1 — Supplementary Information [file 41467_2023_36868_MOESM1_ESM.pdf]

Supplementary Information

Key innovations and the diversification of Hymenoptera

Bonnie B. Blaimer, Bernardo F. Santos, Astrid Cruaud, Michael W. Gates, Robert R. Kula, István Mikó, Jean-Yves Rasplus, David R. Smith, Elijah J. Talamas, Seán G. Brady, Matthew L. Buffington

Correspondence: Bonnie B Blaimer bonnie.blaimer@mfn.berlin

Table of contents

Supplementary Methods **2**
- Taxon sampling 2
- Supplementary Figure 1 3
- Additional phylogenetic inference and data exploration.................... 3
- Extraction of protein-coding UCE sequences 4
- Topology tests using Four-cluster Likelihood Mapping (FcLM) 5
- Divergence dating 5
- Diversification analyses 6
- Character evolution 7
- Trait-dependent and independent diversification 8

Supplementary Discussion **9**
- Data sets and phylogenetic inference 9
- Phylogenetic relationships 10
- Supplementary Table 1 11
- Supplementary Figure 2 12
- GC content filtering 13
- Topology tests using Four-cluster Likelihood Mapping (FcLM) 13
- Supplementary Figures 3–28.................... 15
- Evaluating competing hypotheses on the evolution of Hymenoptera 41
- Supplementary Figure 29 42
- Comparison of divergence dating results 45
- Methodological considerations for macroevolutionary analyses 46

Supplementary References.................... **49**

Supplementary Methods

Taxon sampling and rationale. We assembled a taxon set of 765 species across 94 extant families and all superfamilies of Hymenoptera (Supplementary Data 1). Much of the material used for sequencing came from the vast holdings of the National Museum of Natural History in Washington, DC, or was obtained from other major collections through collaborations. Our access to important legacy collections, combined with the use of genomic ultraconserved elements, allowed us to sequence even old museum specimens and thus sample all major lineages across the root node of each family. In particular, we were able to produce a deep taxonomic sampling of the hyperdiverse superfamilies Ceraphronoidea, Chalcidoidea, Cynipoidea, Ichneumonoidea and Platygastroidea. These groups had only been represented by a few specimens in previous studies focused on the phylogenetic history of Hymenoptera, and their increased representation in our dataset helped us to achieve a more balanced taxon sampling. Our taxon sampling covers from 0.06–50% (=1–150 representatives) of the described species diversity. While we generated UCE sequence data de novo for most taxa, some sequences have already been published in other studies by some of us: 126 aculeate wasps[1,2,3], 25 chalcidoids[4,5,6], 76 cynipoids[7], 26 Ichneumonidae[8,9,10] and 142 Braconidae[11]. We further included six representatives of other insect orders as outgroups by mining UCEs in silico from published genomes: Coleoptera (*Agrilus planipennis*), Diptera (*Aedes albopictus*), Lepidoptera (*Papilio glaucus*), Hemiptera (*Homalodisca vitripennis*), Psocodea (*Pediculus humanus corporis*), Blattodea (*Blattella germanica*). Voucher specimens for newly sequenced taxa are housed at the National Museum of Natural History in Washington, DC. Supplementary Data 1 contains voucher information for all taxa. Specimens sequenced for this study were already accessioned at the National Museum of Natural History in Washington, DC, USA; the Centre de Biologie et de Gestion des Populations, Montpellier, France; the California Academy of Sciences, San Francisco, CA, USA; the Canadian National Collection of Insects, Ottawa, Ontario, Canada; the Florida State Collection of Arthropods, Gainesville, Florida, USA; the National Biodiversity Institute Costa Rica, Costa Rica; the Musée Nationale d'Histoire et Naturelle, Paris, France; the PSU Frost Entomological Museum, Penn State University, PA, USA; and the UCR Entomology Research Museum, Riverside, CA, USA. All specimens have been collected with the required permits and in accordance with local regulations.

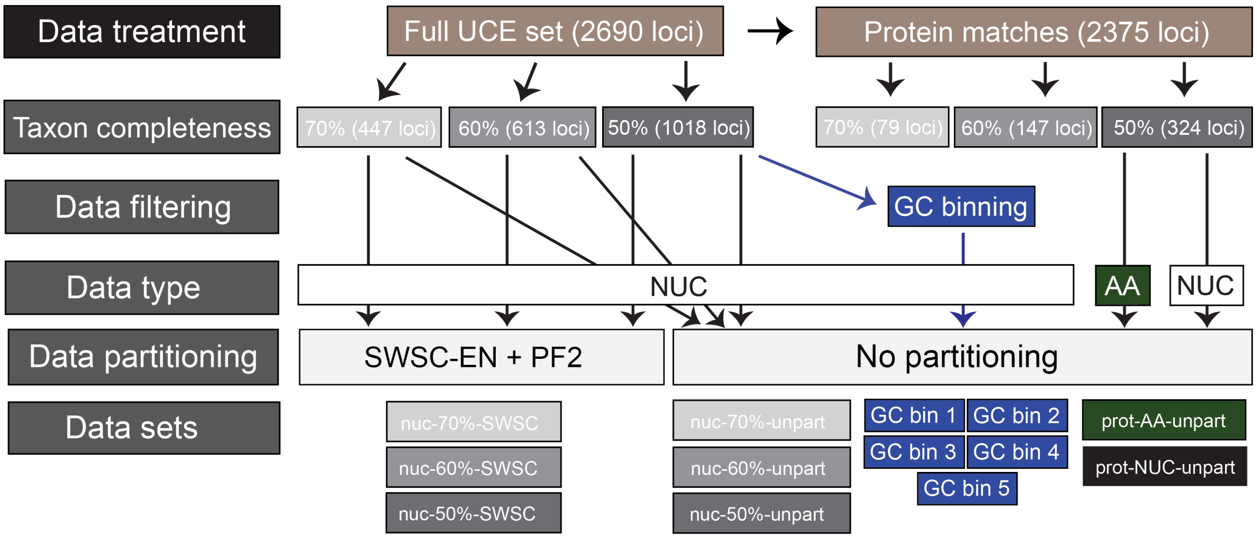

Supplementary Figure 1: Graphical summary of data sets, filtering and partitioning strategies. Taxon completeness filters loci based on the minimum number of taxa represented in the locus alignment file: 70%=539 taxa, 60%=462 taxa, 50%=385 taxa. Further data filtering relied on GC content. Data types were nucleotides (nuc) and amino acids (AA). SWSC-EN = Sliding window site characteristics entropy[12]; PF2 = PartitionFinder2[13].

Additional phylogenetic inference and data exploration. Supplementary Figure 1 gives an overview of all data treatments and analyses. We partitioned the nucleotide data matrices using the Sliding-Window Site Characteristics Entropy (SWSC-EN) algorithm[12], which has been designed specifically to model patterns of rate variation within and among UCE loci by dividing loci into core and flanking regions. We subsequently used PartitionFinder2[13] and the r cluster algorithm[14] to combine subsets with similar properties. We analyzed the resulting partitioned nucleotide data matrices, as well as unpartitioned versions with Maximum Likelihood (ML) best-tree and ultrafast bootstrap searches (with settings: -bb 1000 -wbtl -nt 12 -safe -cptime 60, and -spp for partitioned matrices) in IQ-TREE v1.6.10[15] employing model selection for unpartitioned matrices, while employing a GTR+G model for data subsets in partitioned matrices. The set of 324 protein-coding loci was analyzed both within a nucleotide and an amino acid translated matrix, employing model selection in IQ-TREE but no data partitioning. All analyses were rooted using the outgroup method and *Blattella germanica*. We used the program AMAS[16] to calculate several alignment statistics, e.g., alignment length, amount of missing data, number of parsimony-informative sites (PIC), and base composition of all nucleotide and protein matrices (Supplementary Data 2). We also initially reconstructed gene trees from the 1,118 UCE loci present in the 50% taxon completeness set using SWSC-EN partitioning and IQ-TREE. However, due to the short alignment size of a majority of loci after performing internal trimming with Gblocks and the high amount of missing data, both factors with a high propensity of gene tree estimation errors[17], we did not pursue coalescent analysis with these gene trees.

We investigated variable GC content of UCE loci or sequences as a potential factor leading to uncertainty in reconstructing the evolutionary history of Hymenoptera and particularly the phylogenetic position of Ceraphronoidea and Ichneumonoidea. We calculated GC content per taxon (Supplementary Data 3) and GC content for each UCE locus (Supplementary Data 4) within the 50% matrix using AMAS[16]. We sorted the 1,118 UCE loci with ≥50% taxon completeness into five bins of 224 loci each, based on their GC content (e.g., bin 1 contained the 224 loci with lowest GC content, bin 5 the 224 loci with

highest GC content), concatenated loci in each bin and estimated topologies using unpartitioned ML best tree and bootstrap analyses. Bin size was determined as a trade-off between the goal of capturing the influence of varying GC content between bins while constructing matrices with sufficient phylogenetic information content to generate a resolved phylogeny. Bin membership of UCE loci is presented in Supplementary Data 4.

Since individual UCE loci were very short after applying the relaxed Gblocks trimming (average 118 bp; range 7-483 bp, Supplementary Data 4), we initially also explored several alternative strategies from the above-described alignment and trimming strategies. We first attempted the analysis of locus alignments that were not subjected to internal trimming, however, this resulted in extremely gappy alignments (>97%) and further analyses using untrimmed matrices were therefore abandoned. Next, we implemented MUSCLE[18] (settings: max_divergence=0.2, min-length=100, no-trim) rather than MAFFT as aligner in Phyluce to check whether this program would perform better on our highly divergent sequences. We then applied further relaxed settings in Gblocks to both MUSCLE and MAFFT generated alignments (settings b) b1=0.5, b2=0.5, b3=15, b4=5, and c) b1=0.5, b2=0.5, b3=15, b4=3), and implemented internal trimming with TrimAL (instead of Gblocks) employing the -gappyout method. We performed unpartitioned ML and bootstrap analyses in IQ-TREE from the resulting matrices (70%, 60% and 50% completeness). The more strongly relaxed Gblocks parameters, the TrimAL trimming, and using MUSCLE as aligner instead of MAFFT all resulted in longer alignments (up to almost 2x, with average 224 bp for MUSCLE in combination with Gblocks settings c). However, the resulting topologies were in stark disagreement with well-established hypotheses on the evolution of Hymenoptera, as for example, many of these analyses recovered Ichneumonoidea and Ceraphronoidea as sister lineages to all other Hymenoptera. Both alignment and trimming procedures can have a detrimental effect on information content of phylogenetic data[19]. We concluded from these experiments that our more stringent trimming procedures (as have been employed for UCE analyses in the past) are a necessary trade-off between the benefit of removing hypervariable sites in our data set that seemingly introduce noise due to poor alignment, and the disadvantage of losing potentially valuable phylogenetic information content.

Extraction of protein-coding UCE sequences. To extract protein-coding loci from captured UCEs, we followed a custom-pipeline described in Borowiec[20] and associated script available at https://github.com/marekborowiec/uce-to-protein. The first step of this pipeline requires a reference protein database. We queried NCBI's Taxonomy Browser[21] and downloaded and concatenated all available Hymenoptera protein records (search on April 29, 2019) into one FASTA file and constructed a BLAST database (using uce_to_protein.py blastdb). We obtained a single FASTA file for each UCE locus (all 2590 loci, regardless of taxon completeness) and matched these against our Hymenoptera protein database (uce_to_protein.py queryblast) using BLASTX (https://blast.ncbi.nlm.nih.gov/Blast.cgi). This step results in one BLAST output file per locus for 2375 loci, each containing multiple hits for each UCE sequence that are assigned BLAST scores. These output files were then searched for best scoring hits, which were written to a sqlite database using uce_to_protein.py. This database was then queried using uce_to_protein.py queryprot for all taxa in the data set and unaligned protein-coding sequences were retrieved for each UCE locus. We then aligned these sequences using MAFFT[22] and subsequently trimmed the alignments again using Gblocks[23] with the same settings as indicated above. Filtering of the loci with phyluce_align_get_only_loci_with_min_taxa.py retained 324 loci within a 50% taxon-complete matrix, 147 loci for a 60% complete matrix, and 79 for a 70% complete

matrix. Due to the small size of the 60 and 70% matrices, we then proceeded with phylogenetic analyses only with the 50% complete matrix.

Topology tests using Four-cluster Likelihood Mapping (FcLM). To test support for specific higher-level relationships within Hymenoptera that remained uncertain after phylogenetic estimations, we used the Four-cluster Likelihood Mapping (FcLM) approach[24]. FcLM computes maximum likelihood scores for pre-defined four-cluster splits in a phylogeny based on the phylogenetic information content in an alignment. Phylogenetic support for a particular split, with its three possible resolutions, is visualized graphically by triangular plots with seven divisions (based on seven attractors in the triangle[24]), in which the sequence data are classified according to their information content. The three areas closest the corners of the triangle hereby signify fully resolved relationships (i.e., support for a grouping), the central area equals no support for either possible topology (star-like phylogenies), and the remaining areas are intermediate between two trees[24].

We performed FcLM analyses testing four topological hypotheses (see below) regarding the position of Ichneumonoidea and Ceraphronoidea on each of our four main data sets (nuc-50%, nuc-60%, nuc-70% and prot-AA). The four hypotheses were investigated by defining four taxon groups as specified in Supplementary Data 14. Hypothesis 1 evaluated support from the four data sets for the position of Ichneumonoidea as sister to Ceraphronoidea vs as sister to Proctotrupomorpha (as recovered in topology A-0 vs B); hypothesis 2 evaluated support for the position of Ichneumonoidea as sister to the rest of Apocrita (as recovered by topology C); and hypothesis 3 and 4 evaluated the position of Ceraphronoidea to Aculeata and the Evaniomorpha grade (as recovered by topology C-1 vs C-2). All analyses were run in IQ-TREE v1.6.12 using 100,000 randomly drawn quartets, while employing substitution models estimated by ModelFinder in unpartitioned analyses of the respective data sets.

Divergence dating. We estimated time-calibrated phylogenies using information on twelve fossils of Hymenoptera (Supplementary Data 15), which were chosen to represent the oldest available calibration for superfamily and family-level nodes (with the exception of one representing a subfamily node). We restricted our analysis to these 12 fossils as they covered all major lineages and deep divergences within Hymenoptera for which confidently placed fossils were available. There is evidence that concentrating calibrations on deep nodes increases the accuracy of divergence estimation[25]. Additionally, we set a soft maximum bound of 283.7 Ma for crown Hymenoptera, which represents the upper 95% CI estimated for the age of the order by Misof et al.[26] (see Table S25 in that paper). We initially also implemented a more conservative, older maximum bound of 411 Ma on the Hymenoptera root age, representing the age of the oldest Hexapod fossil, *Rhyniella praecursor*[27]. However, this resulted in the root age and deeper level divergences being pushed unreasonably far back in time, close to this maximum bound, and the age estimates between the two topologies showed wider discrepancies. We therefore decided against pursuing analyses with this older bound on the Hymenoptera root, and here report only results from analyses with the more constrained root calibration. All outgroups were pruned from the tree and alignment prior to divergence time estimation. We employed approximate likelihood to estimate divergence times in MCMCTREE and codeml as included in PAMLv4.9[28], using the two data sets and trees that were best-supported (by ufBS and FcLM) and most frequently recovered across our phylogenetic analyses: (a) the concatenated nuc-50% matrix and the best maximum likelihood tree resulting from SWSC-

EN partitioning of this matrix (topology A-0 = topA-0 hereafter), and (b) the concatenated nuc-70% matrix and the best maximum likelihood tree resulting from SWSC-EN partitioning of this matrix (topology C-1 = topC-1 hereafter). Approximate likelihood calculation is a two-step process consisting first of branch length estimation by maximum likelihood, together with the gradient and Hessian of the likelihood function at the maximum likelihood estimates. Secondly, divergence times are estimated using MCMC sampling and the gradient and Hessian to construct an approximation to the likelihood function. We set up four independent runs each using the independent-rates models and standard parameters. All fossil calibrations were implemented as soft minima, except for the calibration on the root node on which we placed soft minimum and maximum bounds. We used the default settings for the calibration priors (heavy-tailed density based on a truncated Cauchy distribution with an offset p=0.1, a scale parameter c=1 and a left tail probability of α=0.025), as these represent a relatively uninformative distribution appropriate for the almost exclusively stem-group fossil calibrations that were available to us. We realize the caveats going along with uninformative priors (e.g., wide posterior densities), but believe this is preferable to using ones that are informative, but potentially incorrect. We set samplefreq=10 and nsamples=2,000,000, resulting in a potential chain length of 20,000,000 states. We set up four separate runs for each data set, and periodically checked progress and convergence parameters, by visualizing mcmc convergence and effective sample sizes (ESS) using TracerV1.7.1[29]. Runs were stopped at lengths 1,732,010–4,632,960 states once most parameters reached ESS values above 200 (excluding burnin). Most parameters well exceeded the ESS threshold in individual runs, but due to large numbers of parameters to estimate (>750), a few only reached the threshold after combining runs. 731,251 samples for topC-1 and 1,180,127 for topA-0 were summarized across four runs each, after discarding 25–50% of samples as burnin. To evaluate the impact of our calibrations, we also performed analyses without sequence data using only the prior.

Diversification analyses. To assess potential shifts in diversification rates over time in Hymenoptera we used a clade-specific approach in BAMM v2.5[30,31] and the associated R package BAMMtools v2.1.7[32] in R v4.0.3. We used clade-specific sampling probabilities via the sampleProbsFilename element in the BAMM configuration file to account for incomplete sampling. Sampling and parameter choice are further discussed in the Supplementary Discussion (Methodological considerations for macroevolutionary analyses). We assembled a richness matrix for family diversity in Hymenoptera using numbers of described species published in Huber[33], except for the following groups for which the classification in that volume was outdated and did not correspond with natural monophyletic groups. For Bradynobaenidae, Myrmosidae, Thynnidae, Chyphotidae and Tiphiidae, we used the classification established in Pilgrim et al.[34] and species diversity estimates from Branstetter et al.[35]. For specoid wasps we applied the classification of Sann et al.[36] and species estimates listed in Pulaski's catalogue[37]. Within Cynipoidea, we distinguished six monophyletic clades as identified in Blaimer et al.[7] (Cynipidae sensu stricto (s.s.), Figitidae sensu lato (s.l.), Pediaspedini, Paralaucini, Diplolepidini and Eschatocerini) and assigned species richness based on Buffington et al.[38]. Due to the existence of several non-monophyletic families in the Chalcidoidea (e.g., Pteromalidae) and the associated uncertainty about lineage-specific species richness, we treated this entire superfamily as one clade for the purpose of this analysis. We were not able to incorporate the updated taxonomy of Platygastroidea (a split into seven families[39]) into our analyses, therefore this group was also treated as one clade for the purpose of our analyses. The new platygastroid

families are included in the overall count of recognized families and labeled in Fig. 1, however. This merging and splitting of families into recognized monophyletic lineages resulted in 68 clades being defined for analyses. Clade-specific sampling probabilities were then calculated as proportions of sampled diversity divided by total species diversity (refer Supplementary Data 16). Thirteen putative clades (15 missing families, but two were lumped into Chalcidoidea and Platygastroidea for the analyses) were not represented in our phylogeny; therefore, we applied a backbone sampling fraction of 0.8395 (68 sampled clades/81 recognized clades). We used the two chronograms (topA-0 and topC-1) generated in the dating analyses for two sets of BAMM analyses, after using force.ultrametric in phytools v0.7-70[40] as both trees failed a test for ultrametricity. Our analyses were configured using the function "setBAMMpriors" within BAMMtools to obtain appropriate priors for speciation-extinction analyses as outlined in the guidelines in the BAMM documentation (http://bamm-project.org/). The priors used for the analysis were expectedNumberOfShifts=1.0; lambdaInitPrior= 4.98132668558192 (topC-1) and 4.95360853344158 (topA-0); lambdaShiftPrior=0.00411137898883461 (topC-1) and 0.00413438440550203 (topA-0); muInitPrior=4.98132668558192 (topC-1) and 4.95360853344158 (topA-0). We used "speciationextinction" as a model. Our runs included four mcmc chains with a length of 200 million generations, sampling every 10,000 generations, and discarding a burnin of 10%. We confirmed that ESS values were appropriate (>200) and used computeBayesFactors to identify the best supported model of rate shifts in our data. Results were analyzed and plotted with various functions in BAMMtools. We plotted a mean phylorate plot (plot.bammdata), the best shift configuration (getBestShiftConfiguration), the credible shift set (credibleShiftSet) and the cumulative shift probability tree (cumulativeShiftProbsTree). Cumulative shift probabilities were displayed within a range of 0.97–1.00; a less stringent cutoff (e.g., 0.95) supports shifts leading to almost every major clade in the tree, including where these are not present in the best shift configuration (at least for topA-0). Mean speciation and extinction rates were further computed for clades for which rate shifts were indicated by BAMM and MEDUSA (see below).

We further explored diversification rate within our data set using the function MEDUSA[41] in the R package Geiger v2.0.7[42] in R v4.0.3. The MEDUSA algorithm first fits a single diversification model to the entire dataset, and then adds single breakpoints (i.e., shifts) in the diversification process in a stepwise fashion, so that different parts of the tree are allowed to evolve with different parameter values. We implemented this analysis using clade-level chronograms for both topA-0 and topC-1, which we created by dropping all tips except one representative for each of the 68 clades also designated in BAMM analyses. A species richness matrix was further designated, composed of the estimated species diversity for each clade (number of described species; Supplementary Data 16). We used an AICc threshold (3.760758; computed automatically by MEDUSA) as a stopping criterion for the algorithm, at which further breakpoints in the diversification process are not added. We implemented the option cut=node to prevent shifts from being assigned at single terminal branches.

Character evolution. We used the clade-level approach (see above) to score (1) the presence or absence of parasitoidism as a binary trait, and (2) the evolution of hymenopteran life strategies on a more detailed level, assigning the four categories parasitoidism, primary phytophagy (including xylophagy and mycophagy), secondary phytophagy (i.e., gall-inducing, pollen collecting) and predation (Supplementary Data 16).

Moreover, we also (3) contrasted carnivory (parasitoidism and predation combined) with primary and secondary phytophagy in an analysis comprising three trait categories. Clades were assigned to one of these categories based on the life strategy exhibited by the overwhelming majority of members (>80–95%). Kleptoparasitism was not considered as an independent strategy, as on the family-level, there are no taxa that consist entirely, or even to a majority of kleptoparasites. Kleptoparasites are therefore generally lumped with the strategy of the host and their food resource. We allowed polymorphism for Chalcidoidea, Formicidae and Vespidae, for which such a fully binary choice would not be representative of the group. For presence vs absence of parasitoidism, we thus coded 40 clades as parasitoids, 23 clades as non-parasitoid, 1 clade as both parasitoid and non-parasitoid, and four clades as uncertain; for life strategies, we coded 40 clades as parasitoid, 2 clades as predatory, 8 clades as (primary) phytophagous, 11 clades as secondarily phytophagous, 1 clade as parasitoid and secondarily phytophagous, 2 clades as predatory and secondarily phytophagous and four clades as uncertain; for carnivory vs phytophagy, we coded 42 clades as carnivorous, 8 clades as (primary) phytophagous, 11 clades as secondarily phytophagous, 3 clades as carnivorous and secondarily phytophagous, and 4 clades as uncertain (Supplementary Data 16). We used the rayDISC function in the R package corHMMv2.5 in R v4.0.3 to reconstruct ancestral states for presence/absence of parasitoidism and modes of parasitoidism, using both topA-0 and topC-1 (clade-level phylogenies created for MEDUSA analyses). We performed reconstructions under the “equal rates” model (ER) and the “all rates different” model (ARD), comparing the fit of these models by performing a likelihood ratio test on –lnL scores as (1-pchisq(Δ lnL, df)).

Trait-dependent and independent diversification. We tested for state-dependent diversification associated with the four putative key innovations (wasp waist, stinger, parasitoidism and secondary phytophagy) and with alternative life strategies (carnivory, phytophagy (primary + secondary)) in Hymenoptera using the HiSSE (Hidden State Speciation and Extinction) framework and associated R package[43] (v1.9.18). These models have been developed to incorporate hidden states, unobserved traits that influence diversification rate together with the observed traits[43]. In a HiSSE model where the focal states are, for example, 1=parasitoidism present and 0=parasitoidism absent, the diversification parameters of the hidden state will be modeled as a second character with states A and B. This framework addresses concerns about false positive inference of state-dependent diversification when applying less complex models such as BiSSE[44, 45]. We compared 30 models of diversification and trait evolution in the HiSSE framework (Supplementary Data 10), using the full set of models tested by Beaulieu and O’Meara[46], expanded by six models suggested in the HiSSE documentation and by a study assessing the diversification of Squamates[47]. Thus, we tested the fit of our data to a full HiSSE model with unconstrained parameters and 17 subsets hereof with various constraints on transition and diversification rates. Four models were included that excluded hidden states and modeled trait-dependent diversification in a BiSSE-like fashion. Eight character-independent null models, or CID-2 and CID-4 models (sensu Beaulieu & O’Meara[46]), were further tested, also including an extension of the currently implemented CID-4 models in the HiSSE package allowing for nine transition rates[47]. Specifications of model parameters are given in Supplementary Data 10; refer also to Beaulieu and O’Meara[46] and Harrington and Reeder[47]. We performed all analyses using the full phylogenies for topA-0 and topC-1 (same trees as used for BAMM). Calculating the occurrence of these traits across our sampling of Hymenoptera (765 of 152,691 described species), we employed sampling fractions for

character states as follows (absence=0; presence=1): stinger: 0=0.007354953 (n=645) and 1=0.001846296 (n=120); wasp waist: 0=0.003679269 (n=29) and 1=0.005082557 (n=736); parasitoidism: 0=0.00261036 (n=123) and 1=0.00608122 (n=642); carnivory: 0= 0.00344305 (n=100), 1=0.00537821 (n=665); phytophagy: 0=0.00601913 (n=504), 1=0.00378491 (n=261); secondary phytophagy: 0=0.00580169 (n=531), 1=0.00382565 (n=234) (Supplementary Data 17). Since the HiSSE function does not allow for polymorphic states or missing data, we resolved all polymorphisms to the "present" state, and taxa with uncertain states were coded with the state present if that was the case for their closest relatives. This follows the logic that a state can generally be counted as present in a clade even if not present in every taxon, and closely related taxa have a higher probability to share the same traits. We plotted and summarized HiSSE diversification rate and trait reconstruction results for the best-scoring models.

We also employed the MiSSE model, which is a trait-free extension of the HiSSE framework in the same R package (but using version 2.1.9, in R v4.2.2) to focus only on the impact of the unobserved, hidden states on the diversification dynamics of a clade[48]. We used the MiSSEGreedy function and possible.combos=generateMiSSEGreedyCombinations() to automate the process of fitting MiSSE models and the default for stop.deltaAICc=10. We defined a sampling fraction of 0.005 based on our sampling of Hymenoptera (765 of 152,691 described species) and performed analyses using both phylogenies topA-0 and C-1. We summarized results for the best-scoring models in Supplementary Data 13 and visualized the diversification patterns of the hidden states by plotting.

Supplementary Discussion

Data sets and phylogenetic inference. From libraries enriched for UCEs, we were able to generate 10,718–11,761,611 reads per taxon (average: 1,409,467 reads), which assembled into 235–275,787 contigs (average: 50,057 contigs) with an average length of 227–1,094 bp per taxon (average across taxa: 337 bp). The assembled contigs matched 103–2,273 UCE loci (average across taxa: 1,116), with an average length of 234–1,378 bp per taxon (average across taxa: 501 bp). The results of this sequencing effort per taxon are detailed in Supplementary Data 1, and several descriptive statistics from our various alignments are summarized in Supplementary Data 2. Filtering of all captured UCE loci for taxon completeness yielded 1,118 loci in a 50% complete matrix (nuc-50%), 765 loci in a 60% complete matrix (nuc-60%) and 146 loci in a 70% matrix (nuc-70%). The concatenated nuc-50%, nuc-60% and nuc-70% matrices consist of 132,042 bp, 76,942 bp, and 40,672 bp, respectively. Partitioning with the SWSC-EN algorithm[12] and PartitionFinder2[13] resulted in 1018, 613, 325 partitions, for the 50%, 60% and 70% matrices, respectively. Model choice for partitioned matrices was restricted to GTR+G; for models chosen for unpartitioned matrices under unrestricted model choice see Supplementary Data 2. Matching of UCE sequences to a reference protein database and subsequent filtering of the amino acid translated protein-coding loci for taxon completeness retained 324 loci within a 50% taxon-complete matrix. The trimmed concatenated prot-AA-50% matrix consists of 13,198 amino acids.

Phylogenetic relationships. All analyses showed strong support for the Proctotrupomorpha clade including the superfamilies Chalcidoidea, Diaprioidea, Proctotrupoidea, Cynipoidea and Platygastroidea (ultrafast bootstrap support (ufBS)=95–100, across the analyses summarized in Fig. 1 and Supplementary Fig. 2a–f). Chalcidoidea and Mymarommatoidea (*Mymaromma*) were mostly supported as sister taxa (ufBS=100), with Diaprioidea as sister to both (ufBS=100, Supplementary Fig. 2a–e) or sister to Chalcidoidea (ufBS=95, Supplementary Fig. 2f). Proctotrupoidea groups as sister to the Chalcidoidea-Diaprioidea clade with usually strong support (ufBS=100, Supplementary Fig. 2a–e), except in the analysis of the amino acid translated protein–coding data (prot-AA-unpart; Supplementary Fig. 2f). *Ropronia* (Roproniidae) was recovered as a separate lineage from the remaining Proctotrupoidea *s.s.* (*sensu* Sharkey[49]). Cynipoidea and Platygastroidea are further fully supported as sister taxa in most analyses (ufBS=100), except in the prot-AA-unpart analysis (Supplementary Fig. 2f). Trigonaloidea + Megalyridae were recovered as sister to Aculeata, and Evanioidea and Stephanoidea are closely related to the former. The exact relationships of Evanioidea and Stephanoidea to (Trigonaloidea + Megalyridae) + Aculeata varied to some degree due the position of Ceraphronoidea (and Ichneumonoidea), which are unstable between analyses. Relationships of the sawfly lineages were somewhat variable between analyses, but all analyses recover Orussoidea as sister taxon to Apocrita with ufBS=100 (Supplementary Fig. 2a–f), corroborating Vespina (sensu Rasnitsyn 1988). We further see strong support for a single origin of the endophytic sawfly and woodwasp lineages (Unicalcarida), with Siricidae, Xiphydriidae and Cephidae being more closely related to Orussidae + Apocrita than the remaining "symphytan" lineages. Tenthredinoidea or Tenthredinoidea + (Xyeloidea + Pamphilioidea) are estimated as the sister group to all other Hymenoptera (Fig. 1, Supplementary Fig. 2a–f). Full results of all analyses are shown in Supplementary Figs 3–15.

We recovered three competing hypotheses concerning the position of Ichneumonoidea, in combination with three alternative positions of Ceraphronoidea. We summarized these by establishing mutually exclusive topology groupings based on the superfamily-level relationships, where letters (A–C) specify the position of Ichneumonoidea and numbers (0–2) specify the position of Ceraphronoidea (Supplementary Table 1). Best supported with regard to ufBS and FcLM support for superfamily-relationships is topology C (Supplementary Fig. 2a–b), where Ichneumonoidea groups as the sister to all Apocrita, while Ceraphronoidea is estimated as sister to Evanioidea + Stephanoidea (Supplementary Fig. 2a; C-1: nuc-70%-SWSC), or, with less support, as sister to Stephanoidea (Supplementary Fig. 2b; C-2: nuc-70%-unpart). The most frequently recovered topology (i.e., the topology recovered in a larger number of individual analyses) is topology A-0, in which Ichneumonoidea and Ceraphronoidea are forming a weakly to moderately supported clade (ufBS=75–94) that is sister to the Proctotrupomorpha (ufBS=75–92) (Supplementary Fig. 2c–e). This grouping is recovered by several data sets: nuc-50%-SWSC and nuc-50%-unpart (Supplementary Fig. 2c), nuc-60%-SWSC and nuc-60%-unpart (Supplementary Fig. 2d) and prot-NUC-unpart (Supplementary Fig. 2e). Topology B-1 was supported by analysis of the amino acid translated protein-coding data (prot-AA-unpart, Supplementary Fig. 2d), which recovers Ichneumonoidea as sister to Proctotrupomorpha (ufBS=93), and Ceraphronoidea as sister to Evanioidea and Stephanoidea (ufBS=94).

Despite the uncertainty around the position of Ichneumonoidea and Ceraphronoidea, we recovered full support for two important subdivisions within Apocrita, consisting of (1) the Proctotrupomorpha and (2) a clade consisting of Aculeata and the former "Evaniomorpha" (itself a grade). The ambiguous positions of the two superfamilies are further shown to have

little consequence to reconstructing life histories and the diversification history of Hymenoptera.

Supplementary Table 1: Major hypotheses on the positions of Ichneumonoidea and Ceraphronoidea recovered by our analyses.

Position of Ichneumonoidea	Position of Ceraphronoidea		Analyses
**A**: sister to Ceraphronoidea	**0**: sister to Ichneumonoidea	**A-0**	nuc-50%-SWSC, nuc-50%-unpart, nuc-60%-SWSC, nuc-60%-unpart, Prot-NUC-unpart
B: sister to Proctotrupomorpha	1: sister to Evanioidea + Stephanoidea	B-1	Prot-AA-unpart
**C**: sister to rest of Apocrita	**1**: sister to Evanioidea + Stephanoidea	**C-1**	nuc-70%-SWSC
C: sister to rest of Apocrita	2: sister to Stephanoidea	C-2	nuc-70%-unpart

Summary of the superfamily-level topologies recovered by main analyses, as shown in Supplementary Figure 2. The two most supported combinations are highlighted in bold font. For a detailed description of data sets and analyses, refer to Supplementary Methods and Supplementary Data 2 and 4.

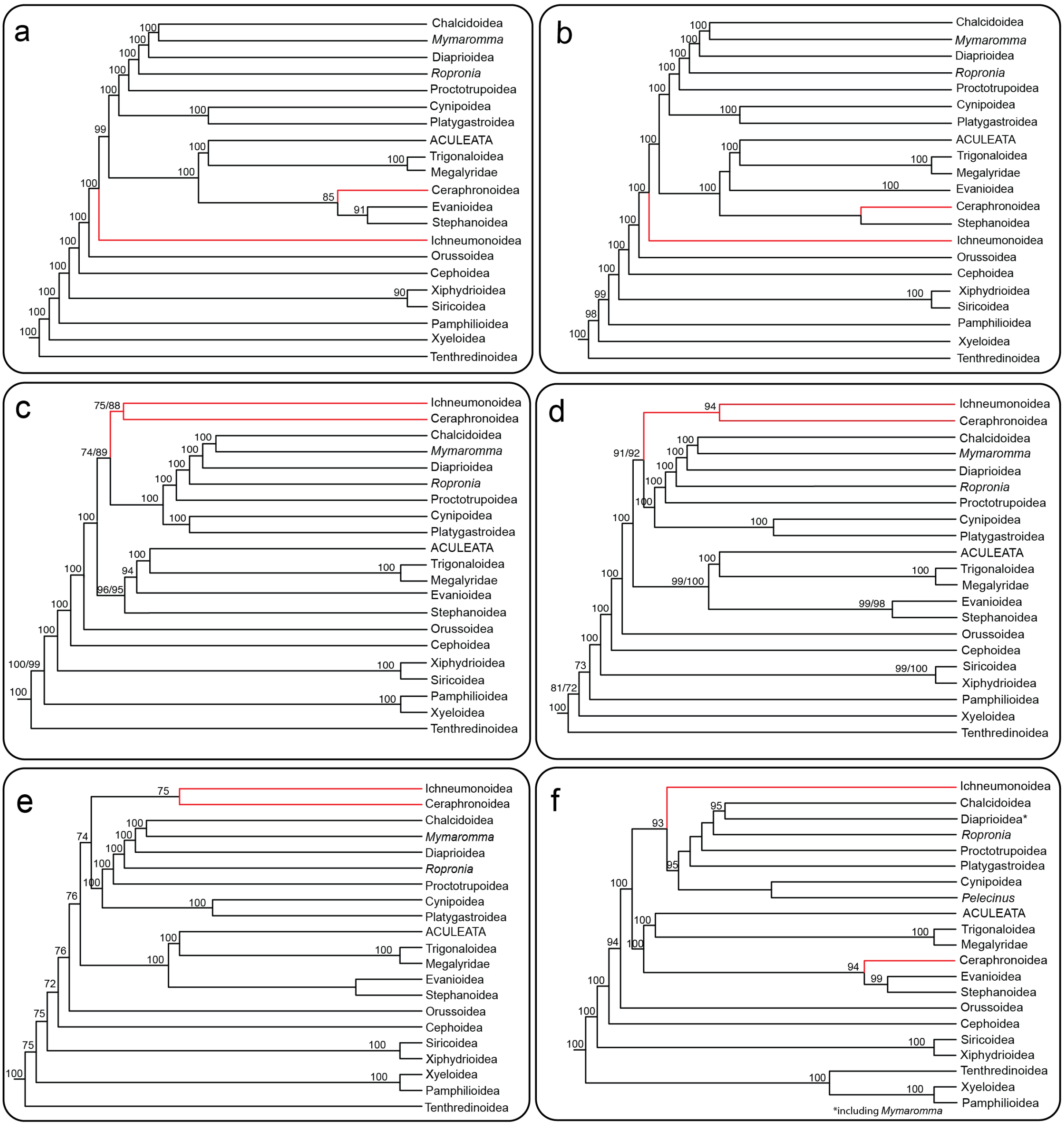

Supplementary Figure 2: **Super-family level summaries of competing topologies supported by main analyses**. Summary trees as estimated from several arrangements of the entire dataset with varying levels of missing data and phylogenetic information content, partitioned vs unpartitioned strategies, and using Maximum Likelihood (ML) best-tree and ultrafast bootstrap in IQ-TREE v1.6.10[15]. **a: Topology C-1**: Ichneumonoidea sister to rest of Apocrita (incl. Ceraphronoidea), Ceraphronoidea sister to Evanioidea + Stephanoidea; depicted is topology and support resulting from nuc-SWSC-70% analysis; **b: Topology C-2**: Ichneumonoidea sister to rest of Apocrita + Ceraphronoidea, Ceraphronoidea sister to Stephanoidea; depicted is topology and support resulting from nuc-unpart-70% analysis; **c: Topology A-0**: Ichneumonoidea sister to Ceraphronoidea; depicted is topology resulting from nuc-SWSC-50% analysis, with support values also summarizing across nuc-unpart-50% analysis; **d: Topology A-0**: Ichneumonoidea sister to Ceraphronoidea; summarized is support from nuc-SWSC-60% and nuc-unpart-60% analyses; **e: Topology A-0**: Ichneumonoidea sister to Ceraphronoidea; depicted is support from Prot-nuc-unpart analysis; **f: Topology B-1**: Ichneumonoidea sister to Proctotrupomorpha, Ceraphronoidea sister to Evanioidea + Stephanoidea; depicted is topology and support resulting from Prot-AA-unpart analysis. Non-Hymenopteran outgroups have been removed from the summary trees. Branches leading to Ichneumonoidea and Ceraphronoidea are highlighted in red. Support values below ufBS=70 are not shown. Source data for this figure can be found in the Dryad repository at https://doi.org/10.5061/dryad.08kprr54m (folder 2.1)[86].

GC content filtering. The GC content analyzed across taxa varied between 0.38–0.52; however, average GC content between superfamilies was similar (0.44–0.50) and did not indicate a bias within particular groups of taxa (Supplementary Data 3). In contrast, the GC content varied greatly across loci, from 0.15–0.75 (Supplementary Data 4). We therefore sorted UCE loci into bins based on their GC content and analyzed these separately to test for potential GC bias in phylogenetic inference. The inferred ML trees are shown in Supplementary Figs 11–15. Although topologies B and C recovered in the main analyses were also represented by some of the binned analyses, we did not observe a trend where one topology was recovered exclusively by bins with either high or low GC content, which would indicate a systematic bias among loci based on GC content. Topology B was recovered by loci with low GC content (GC-bin 1) and high GC content (GC-bin 4), albeit with a variation where Ceraphronoidea groups as sister to Stephanoidea (Supplementary Fig. 11), or as sister to a clade containing Aculeata, Trigonaloidea, Megalyridae, Evanioidea and Stephanoidea (Supplementary Fig. 14). The bin with highest GC content (GC bin 5) supported topC-1 (Supplementary Fig. 15), which was a result also recovered by the nuc-70% matrix. The locus composition of GC bin 5, however, only showed partial overlap with the nuc-70% matrix (Supplementary Data 4), and GC content of the latter was not elevated compared to the other main matrices (Supplementary Data 2). Analyses of GC bin 2 and 3 (medium levels of GC content) did not recover either of the topologies generated by main analyses, but gave implausible or outright spurious results, for example, suggesting Ichneumonoidea as sister to all Hymenoptera (Supplementary Fig. 13), or placing an outgroup taxon within the ingroup (Supplementary Fig. 12). These results are most likely artifacts of lower phylogenetic information content in the smaller data subsets, paired with high amounts of missing data in some taxa.

Topology tests using Four-cluster Likelihood Mapping (FcLM). We tested support for four conflicting hypotheses with regard to the positions of Ichneumonoidea and Ceraphronoidea using the Four-cluster Likelihood Mapping (FcLM) approach[24] and the four main data sets (nuc-50%, nuc-60%, nuc-70% and prot-AA). We did not include the prot-NUC data set here as a main data set for FcLM as we regard this only as a variant of the main prot-AA matrix. Hypotheses 1 and 2 primarily investigated the position of Ichneumonoidea, while hypotheses 3 and 4 focused on the position of Ceraphronoidea.

Specifically, Hypothesis 1 evaluated support for the position of Ichneumonoidea as sister to Ceraphronoidea versus Ichneumonoidea being sister to Proctotrupomorpha, relationships that were recovered in topologies A, estimated by nuc-50%-SWSC, nuc-50%-unpart, nuc-60%-SWSC, nuc-60%-unpart and prot-nuc-unpart ML analyses, vs topology B estimated by prot-AA-unpart ML analysis (see Supplementary Table 1). The nuc-50%, nuc-60%, nuc-70% and prot-AA alignments contain 90.7%, 89.5%, 86.4%, and 74.0% decisive quartets regarding this split, respectively. However, support remains almost completely equivocal for each alternative grouping (Supplementary Data 5). A grouping where Ichneumonoidea is sister to Ceraphronoidea (topA-0) is basically equally supported as a grouping where Ichneumonoidea is sister to Proctotrupomorpha (top. B) by the nuc-50% and nuc-60% alignments, despite inferred trees from these two alignments returning highest overall ufBS for topA-0. The nuc-70% alignment renders marginally higher quartet support (34.3%) for topology B (Ichneumonoidea is sister to Proctotrupomorpha), while the prot-AA matrix lends the lowest quartet support for topology B (17.2%), despite this result being supported in ML analyses of this matrix.

Hypothesis 2 evaluated quartet support for Ichneumonoidea being sister to the rest of Apocrita, a result that was originally recovered by topology C, estimated from nuc-70%-SWSC and nuc-70%-unpart analyses. This grouping is best-supported over any alternative solutions by all data sets (nuc-50%: 42.1%; nuc-60%: 43.4%; nuc-70%: 36.1%; prot-AA: 36.5%). Decisive quartets for groupings within this hypothesis range between 89.0–72.9% (Supplementary Data 5).

Hypothesis 3 evaluated the position of Ceraphronoidea as either sister to Evanioidea, Stephanoidea, or Aculeata plus Trigonaloidea and Megalyroidea; decisive quartets for groupings within this hypothesis ranged between 64.7–92.8%. The prot-AA alignment here supports a sister group relationship between Evanioidea and Stephanoidea (41.2% of quartets), while the nucleotide matrices lend highest (relative) support for a sister-group relationship of Ceraphronoidea with Evanioidea (nuc-50%: 57.2%, nuc-60%: 50.9%; nuc-70%: 44.5%), a grouping that had not been recovered in any of the ML analyses.

Hypothesis 4 evaluated the position of Ceraphronoidea as sister to either Evanioidea + Stephanoidea, Aculeata or Trigonaloidea + Megalyroidea. Decisive quartets for groupings within this hypothesis ranged between 78.4–94.4%. Nucleotide matrices lend highest (relative) support to a sister-group relationship of Ceraphronoidea with Evanioidea + Stephanoidea (nuc-50%: 42.8%, nuc-60%: 38.9%; nuc-70%: 50.3%), a grouping which was recovered in nuc-70%-SWSC and prot-AA-unpart analyses. The prot-AA matrix gave highest support (36.2%) to a sister-group relationship between Ceraphronoidea and Trigonaloidea + Megalyroidea.

Overall, these results indicate a substantial amount of conflict in the data, despite high phylogenetic information content in the nucleotide matrices with respect to the phylogenetic hypotheses (nuc-50%: 89.0–94.4%; nuc-60%: 86.4–93.1%; nuc-70%: 82.9–92.5%). The protein-coding data set (prot-AA) demonstrates less phylogenetic signal with 64.7–78.4% decisive quartets. The best quartet support overall was received for Ichneumonoidea grouping as sister to Apocrita (hypothesis 2, 36.1–43.4%) and Ceraphronoidea grouping as sister to Evanioidea + Stephanoidea (hypothesis 4, 38.9–50.3%) (Supplementary Data 5), which are the positions recovered in topology C-1 (Figs 1 and 2a). These results led us to favor topology C-1 for display throughout the main text. However, given the results obtained by testing of Hypothesis 3, a sister-group relationship of Ceraphronoidea to Evanioidea cannot be discounted, albeit this relationship is not recovered otherwise in maximum likelihood analyses.

Supplementary Fig. 3: ML phylogeny estimated from the unpartitioned nuc-50% matrix using best-tree and 1000 ultrafast bootstrap searches in IQ-TREE v1.6. For description of data set, see main and supplementary text and Supplementary Data 2. Source data for this figure can be found in the Dryad repository at https://doi.org/10.5061/dryad.08kprr54m (folder 2.1)[86].

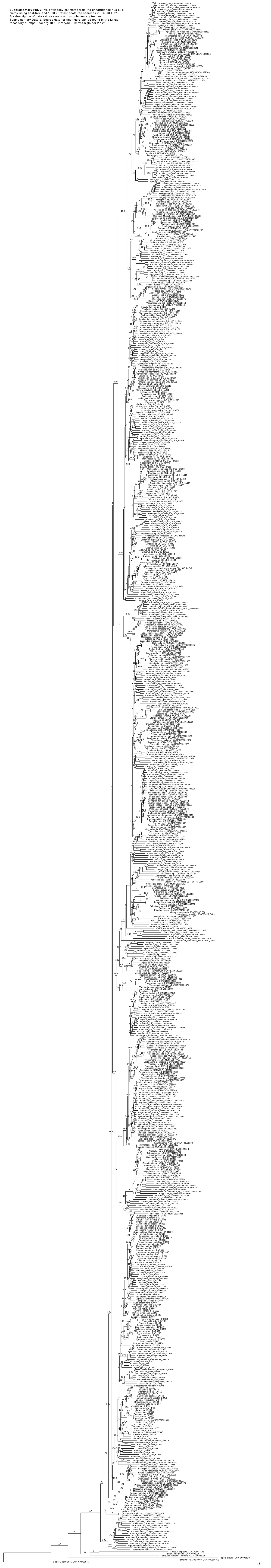

Supplementary Fig. 4: ML phylogeny estimated from the nuc-50% matrix using best-tree and 1000 ultrafast bootstrap searches in IQ-TREE v1.6, employing 1018 partitions selected with the SWSC-EN algorithm and the rcluster algorithm in PartitionFinder2. For description of data set, see main and supplementary text and Supplementary Data 2.Source data for this figure can be found in the Dryad repository at https://doi.org/10.5061/dryad.08kprr54m (folder 2.1)[86].

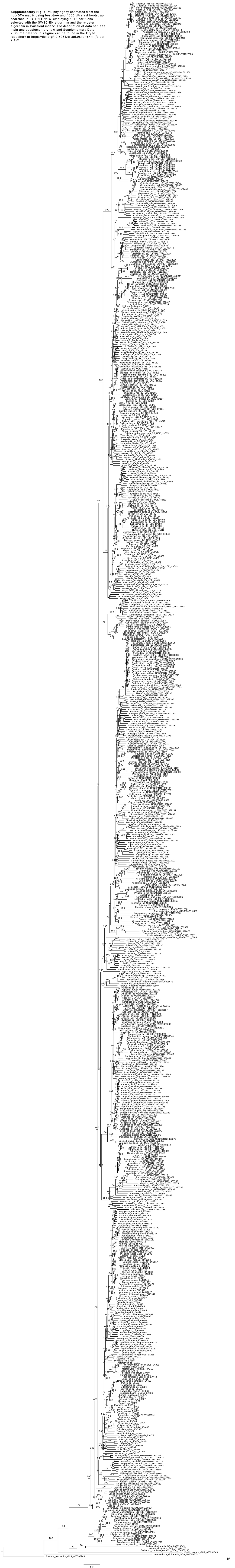

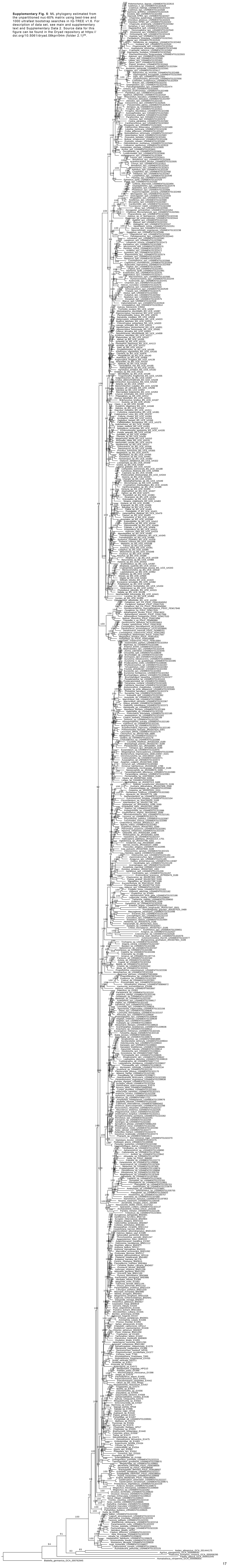

Supplementary Fig. 5: ML phylogeny estimated from the unpartitioned nuc-60% matrix using best-tree and 1000 ultrafast bootstrap searches in IQ-TREE v1.6. For description of data set, see main and supplementary text and Supplementary Data 2. Source data for this figure can be found in the Dryad repository at https://doi.org/10.5061/dryad.08kprr54m (folder 2.1)[86].

Supplementary Fig. 6: ML phylogeny estimated from the nuc-60% matrix using best-tree and 1000 ultrafast bootstrap searches in IQ-TREE v1.6, employing 613 partitions selected with the SWSC-EN algorithm and the rcluster algorithm in PartitionFinder2. For description of data set, see main and supplementary text and Supplementary Data 2. For description of data set, see main text and Supplementary Data 2.Source data for thi figure can be found in the Dryad repository at https://doi.org/10.5061/dryad.08kprr54m (folder 2.1)[86].

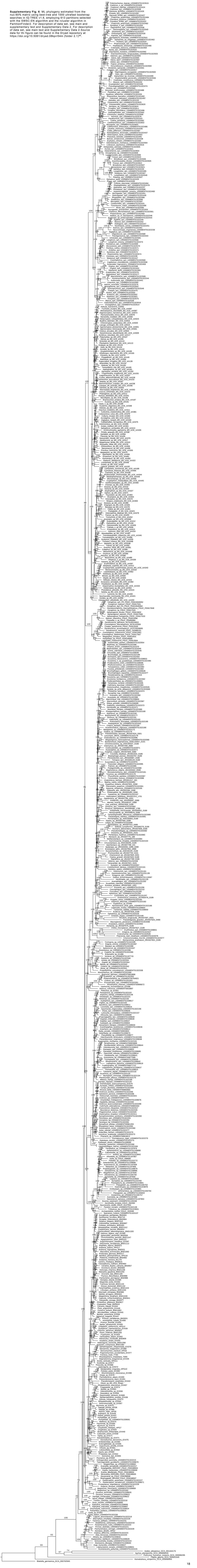

Supplementary Fig. 7: ML phylogeny estimated from the unpartitioned nuc-70% matrix using best-tree and 1000 ultrafast bootstrap searches in IQ-TREE v1.6. For description of data set, see main and supplementary text and Supplementary Data 2. Source data for this figure can be found in the Dryad repository at https://doi.org/10.5061/dryad.08kprr54m (folder 2.1)[86].

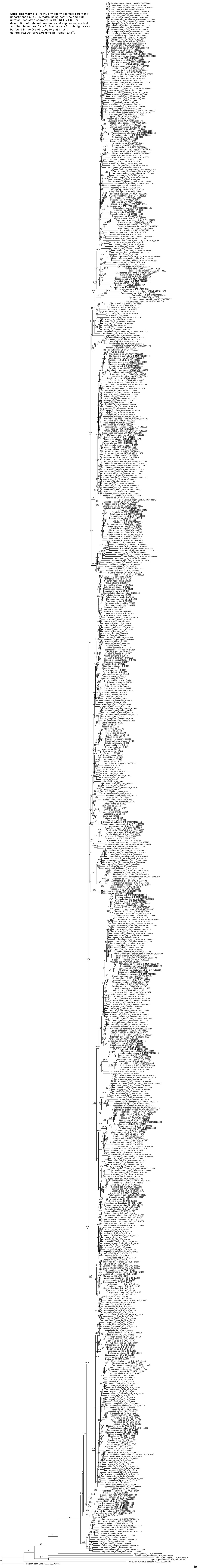

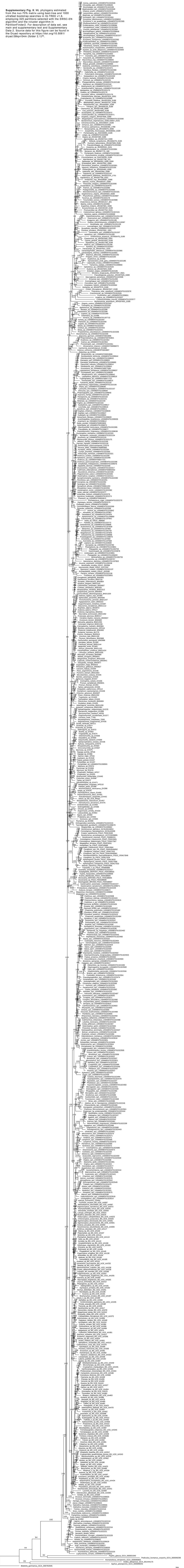

Supplementary Fig. 8: ML phylogeny estimated from the nuc-70% matrix using best-tree and 1000 ultrafast bootstrap searches in IQ-TREE v1.6, employing 325 partitions selected with the SWSC-EN algorithm and the rcluster algorithm in PartitionFinder2. For description of data set, see main and supplementary text and Supplementary Data 2. Source data for this figure can be found in the Dryad repository at https://doi.org/10.5061/dryad.08kprr54m (folder 2.1)[86].

Supplementary Fig. 9: ML phylogeny estimated from the unpartitioned prot-AA matrix using best-tree and 1000 ultrafast bootstrap searches in IQ-TREE v1.6. For description of data set, see main and supplementary text and Supplementary Data 2. Source data for this figure can be found in the Dryad repository at https://doi.org/10.5061/dryad.08kprr54m (folder 2.1)[86].

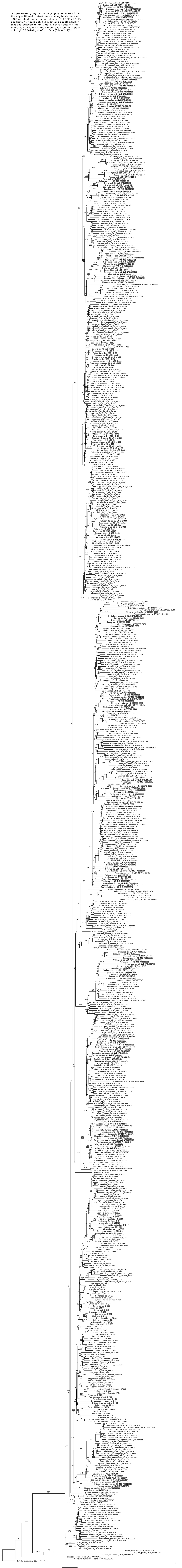

Supplementary Fig. 10: ML phylogeny estimated from the unpartitioned prot-NUC matrix using best-tree and 1000 ultrafast bootstrap searches in IQ-TREE v1.6. For description of data set, see main and supplementary text and Supplementary Data 2. Source data for this figure can be found in the Dryad repository at https://doi.org/10.5061/dryad.08kprr54m (folder 2.1)[86].

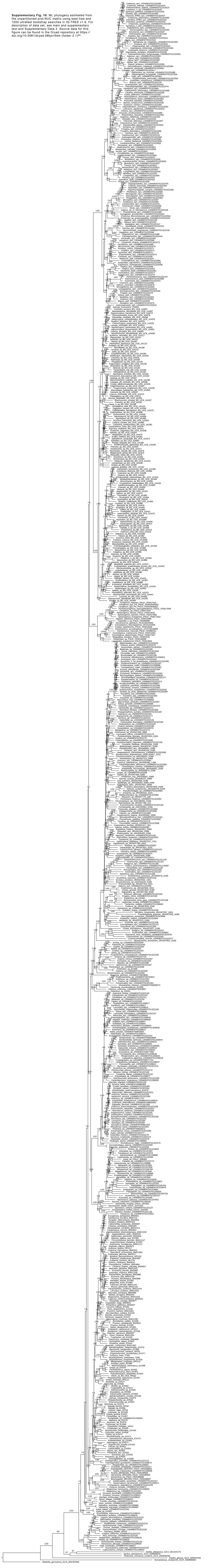

Supplementary Fig. 11: ML phylogeny estimated from GC bin 1 using best-tree and 1000 ultrafast bootstrap searches in IQ-TREE v1.6. For description of data set, see main and supplementary text and Supplementary Data 2.Source data for this figure can be found in the Dryad repository at https://doi.org/10.5061/dryad.08kprr54m (folder 2.1)[86].

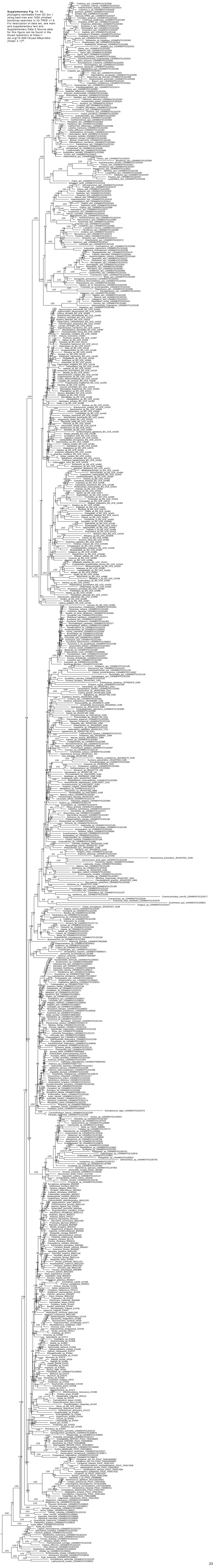

Supplementary Fig. 12: ML phylogeny estimated from GC bin 2 using best-tree and 1000 ultrafast bootstrap searches in IQ-TREE v1.6. For description of data set, see main and supplementary text and Supplementary Data 2. Source data for this figure can be found in the Dryad repository at https://doi.org/10.5061/dryad.08kprr54m (folder 2.1)[86].

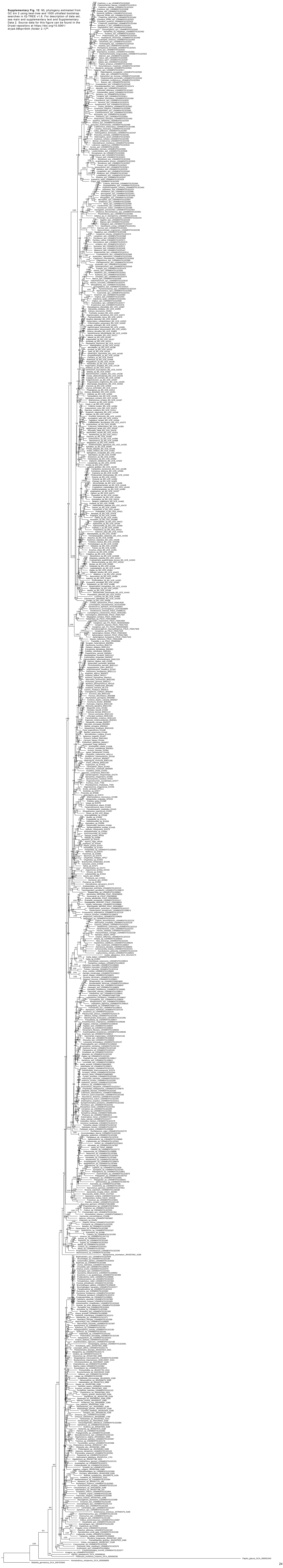

Supplementary Fig. 13: ML phylogeny estimated from GC bin 3 using best-tree and 1000 ultrafast bootstrap searches in IQ-TREE v1.6. For description of data set, see main and supplementary text and Supplementary Data 2. Source data for this figure can be found in the Dryad repository at https://doi.org/10.5061/dryad.08kprr54m (folder 2.1)[86].

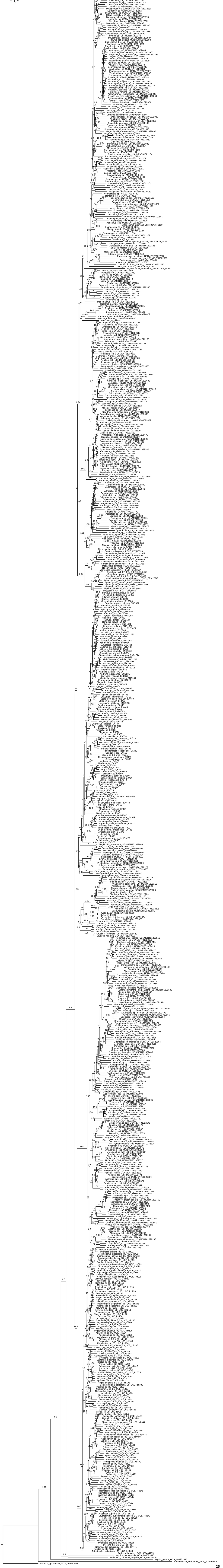

Supplementary Fig. 14: ML phylogeny estimated from GC bin 4 using best-tree and 1000 ultrafast bootstrap searches in IQ-TREE v1.6. For description of data set, see main and supplementary text and Supplementary Data 2. Source data for this figure can be found in the Dryad repository at https://doi.org/10.5061/dryad.08kprr54m (folder 2.1)[86].

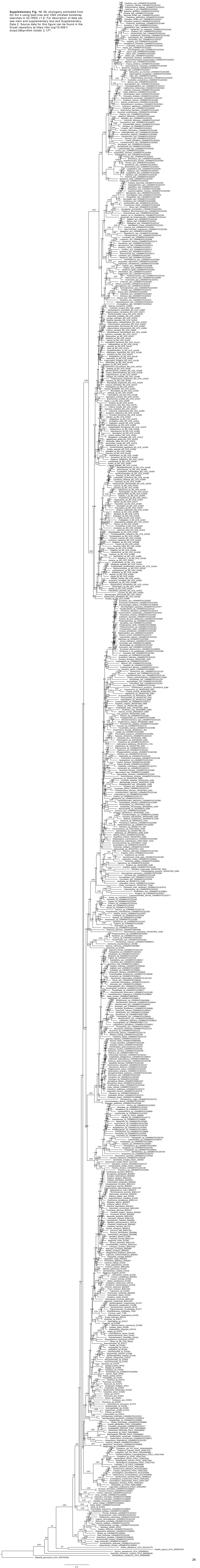

Supplementary Fig. 15: ML phylogeny estimated from GC bin 5 using best-tree and 1000 ultrafast bootstrap searches in IQ-TREE v1.6. For description of data set, see main and supplementary text and Supplementary Data 2. Source data for this figure can be found in the Dryad repository at https://doi.org/10.5061/dryad.08kprr54m (folder 2.1)[86].

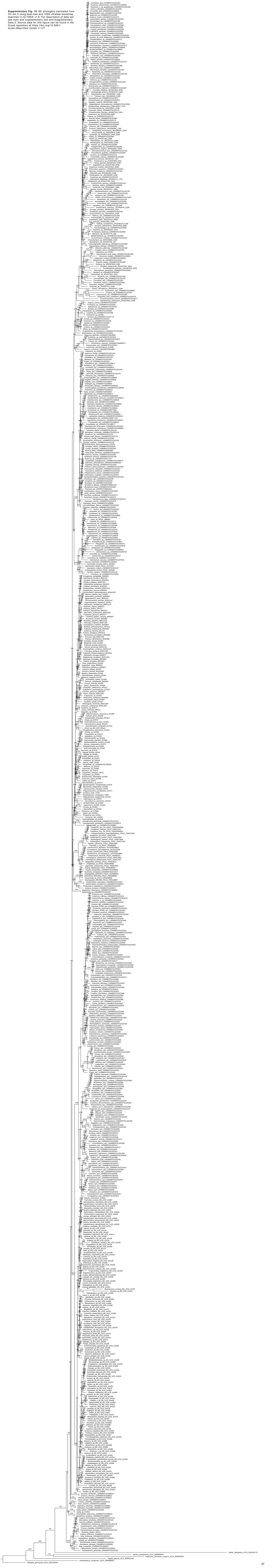

Supplementary Fig. 16: Chronogram estimated from the nuc-70% matrix and topology C-1 resulting from SWSC partitioning of this matrix, using approximate likelihood with mcmctree and codeml as included in PAMLv4.9. Two runs with 5,563,155 combined states were summarized after discarding 25% burnin. Source data for this figure can be found in the Dryad repository at https://doi.org/10.5061/dryad.08kprr54m (folder 2.2)[86].

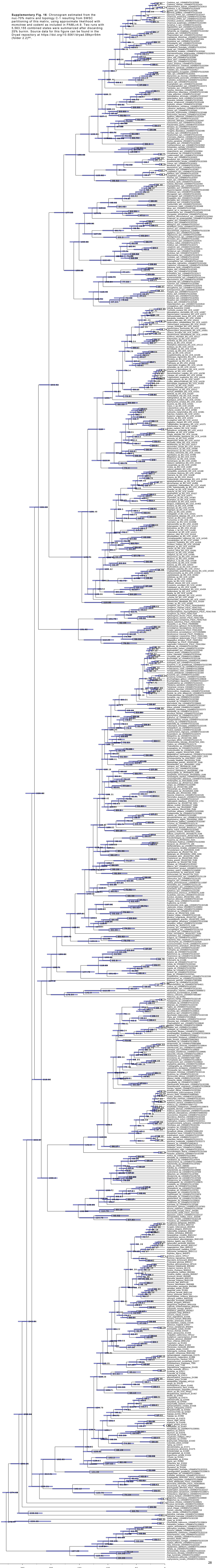

Supplementary Fig. 17: Chronogram estimated from the nuc-50% matrix and topology A-0 resulting from SWSC partitioning of this matrix, using approximate likelihood with mcmctree and codeml as included in PAMLv 4.9. Four runs with 12,727,869 combined states were summarized after discarding 30-50% burnin. Source data for this figure can be found in the Dryad repository at https://doi.org/10.5061/dryad.08kprr54m (folder 2.2)[86].

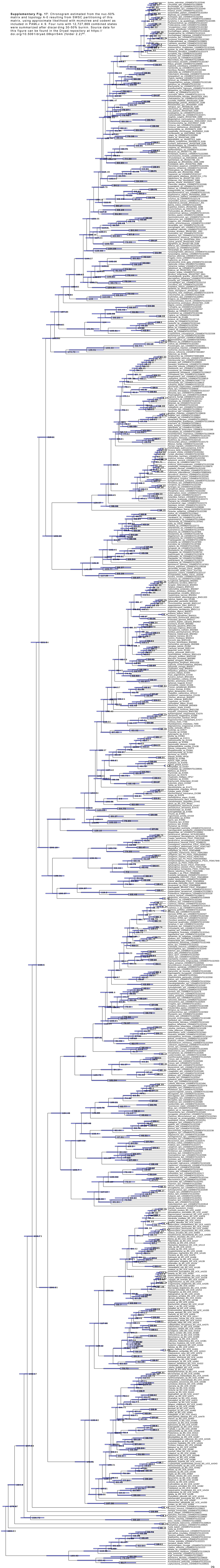

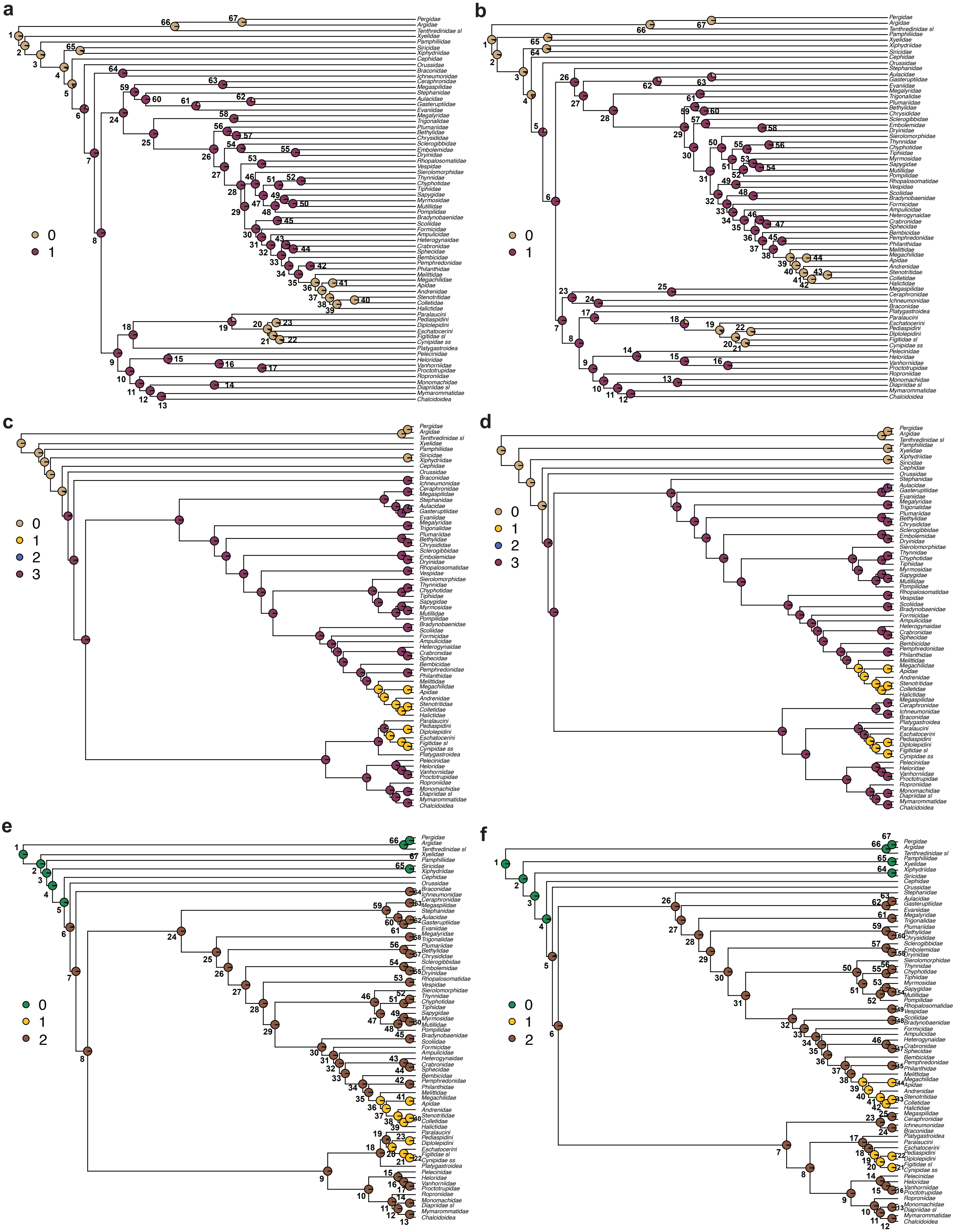

Supplementary Fig. 18: **Additional ancestral character reconstructions under the ER model**. Additional results for ancestral reconstructions. **a**: topology C-1, no parasitoidism (0) vs parasitoidism (1); **b**: topology A-0, no parasitoidism (0) vs parasitoidism (1); **c**: topology C-1, four strategies: primary phytophagy (0), secondary phytophagy (1), predation (2), parasitoidism (3); **d**: topology A-0, four strategies: primary phytophagy (0), secondary phytophagy (1), predation (2), parasitoidism (3). **e**: topology C-1, types of phytophagy: primary phytophagy (0) vs 1=secondary phytophagy (1) vs carnivory (2); **f**: topology A-0, types of phytophagy: primary phytophagy (0) vs 1=secondary phytophagy (1) vs carnivory (2). Node numbers refer to Supplementary Data 8. Source data for this figure can be found in the Dryad repository at https://doi.org/10.5061/dryad.08kprr54m (folder 3.1)[86].

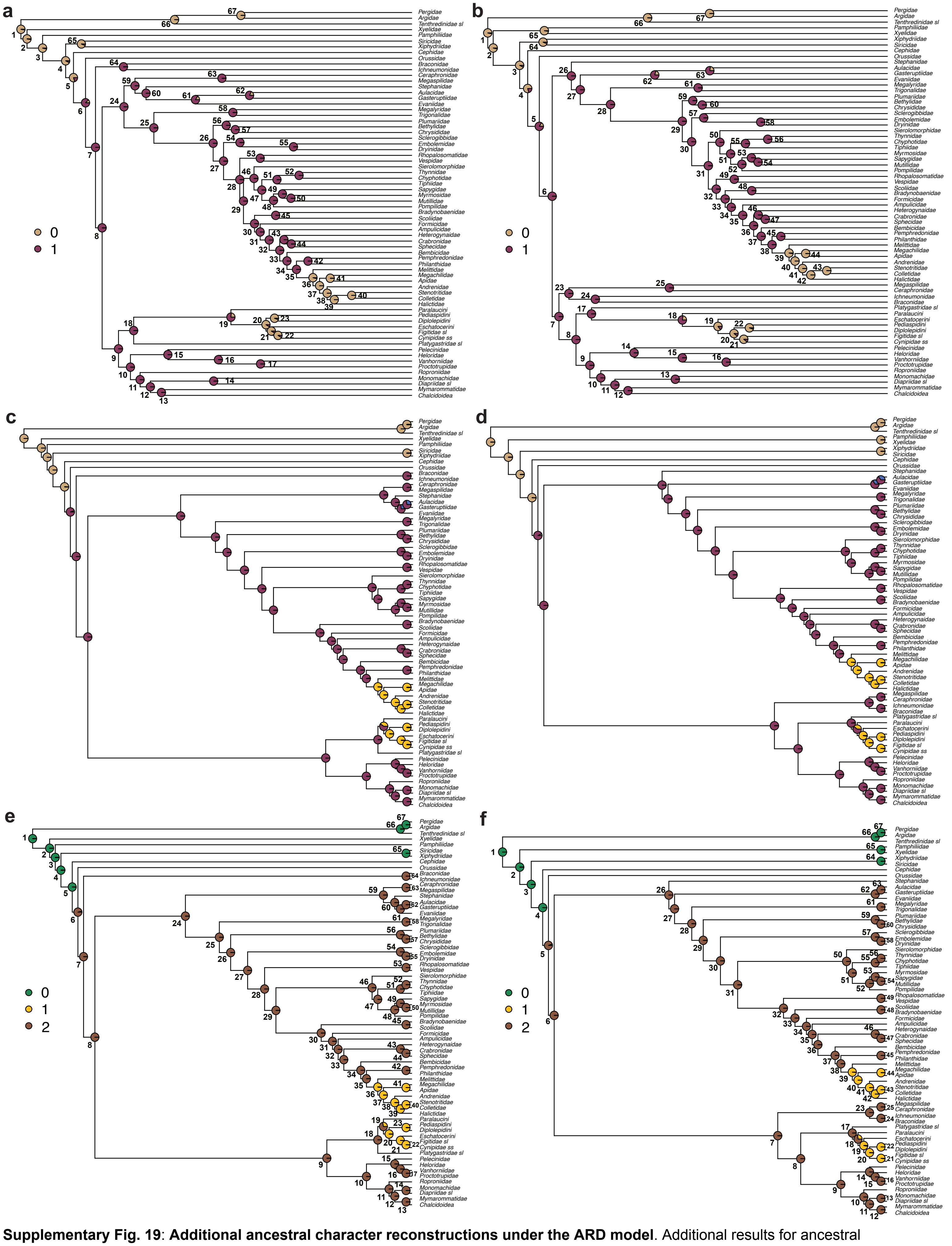

Supplementary Fig. 19: **Additional ancestral character reconstructions under the ARD model**. Additional results for ancestral reconstructions. **a**: topology C-1, no parasitoidism (0) vs parasitoidism (1); **b**: topology A-0, no parasitoidism (0) vs parasitoidism (1); **c**: topology C-1, four strategies: primary phytophagy (0), secondary phytophagy (1), predation (2), parasitoidism (3); **d**: topology A-0, four strategies: primary phytophagy (0), secondary phytophagy (1), predation (2), parasitoidism (3). **e**: topology C-1, types of phytophagy: primary phytophagy (0) vs 1=secondary phytophagy (1) vs carnivory (2); **f**: topology A-0, types of phytophagy: primary phytophagy (0) vs 1=secondary phytophagy (1) vs carnivory (2). Node numbers refer to Supplementary Data 8. Source data for this figure can be found in the Dryad repository at https://doi.org/10.5061/dryad.08kprr54m (folder 3.1)[86].

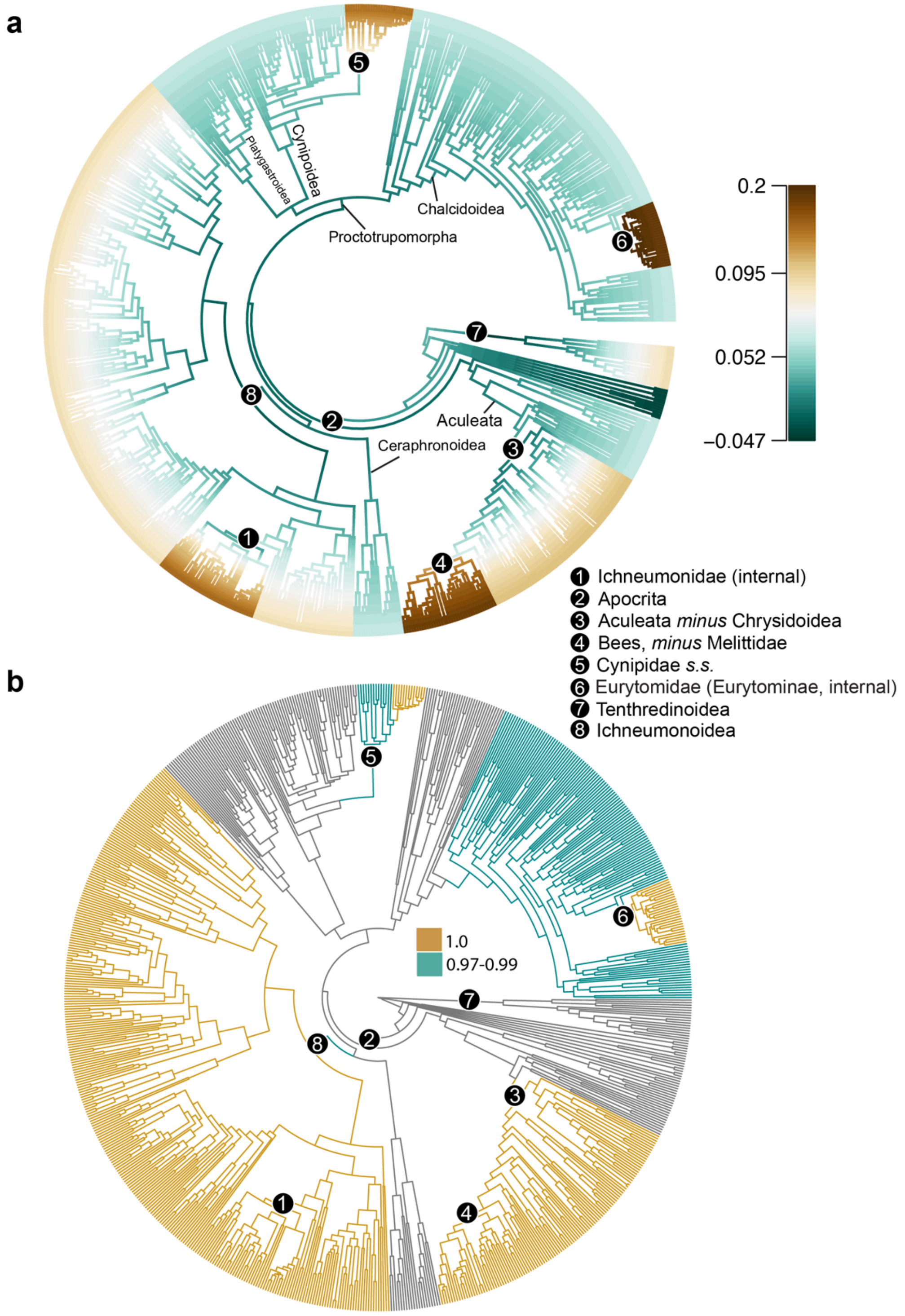

Supplementary Figure 20: **Diversification rate shifts estimated with BAMM from topology A-0**. **a**: Plot of best shift configuration with maximum a posteriori probability (MAP), indicating rate shifts on respective branches. Since this rate shift configuration only has a 0.009 (A-0) probability among 12454 distinct shift configurations in the 95% credibility set, we summarized the cumulative probabilities for each branch that a shift occurred somewhere between the focal branch and the root of the tree. **b**: cumulative shift probability tree, indicating in cyan the branches with a shift probability ≥0.97≥0.99 and in gold the branches with a cumulative shift probability =1. Golden branches thus occur in every distinct shift configuration, cyan branches in 97–99% of all distinct shift configurations. Source data for this figure can be found in the Dryad repository at https://doi.org/10.5061/dryad.08kprr54m (folder 3.2)[86].

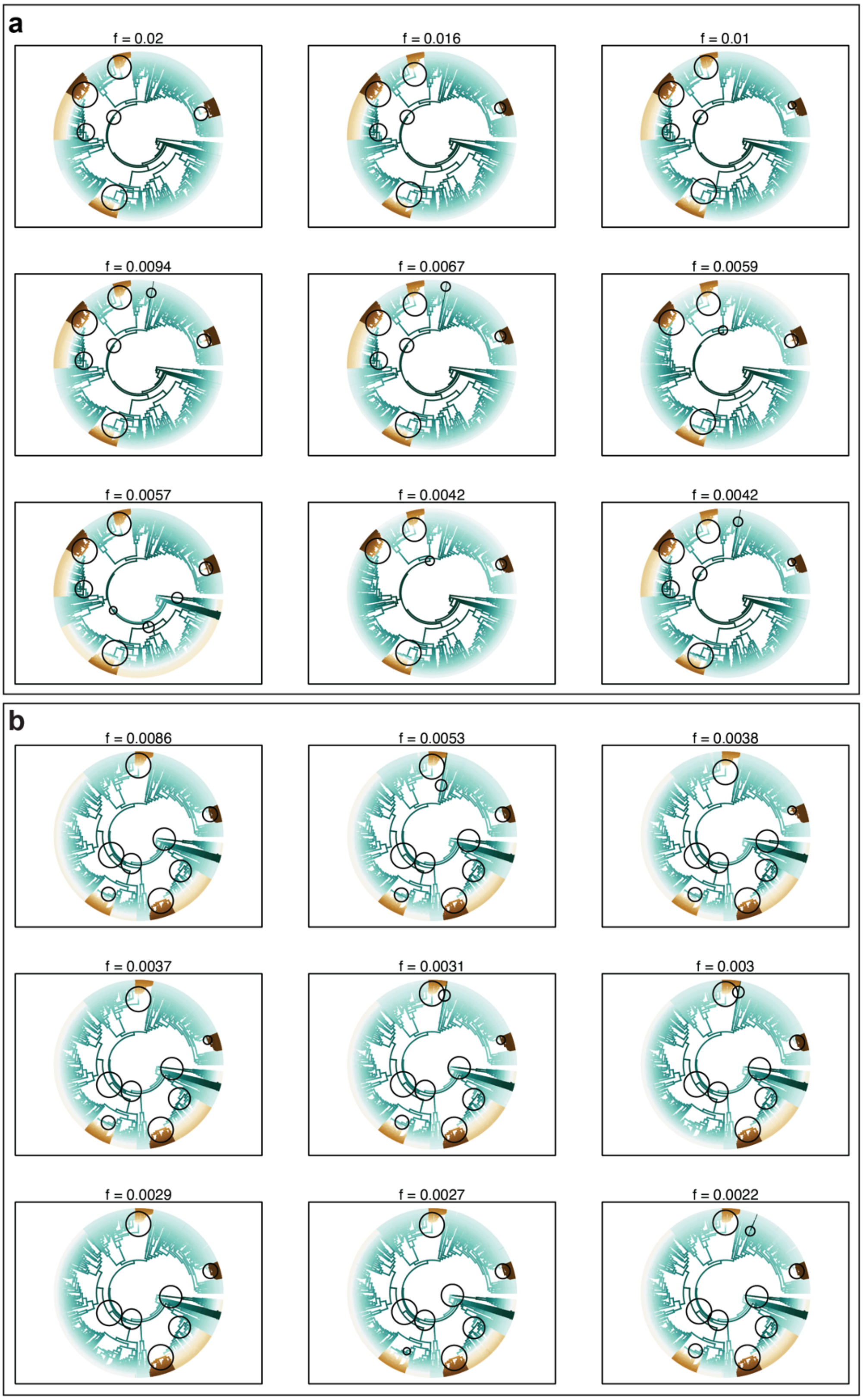

Supplementary Figure 21: **Most frequent shift configurations estimated by BAMM**. **a:** Plots of the 9 most frequent shift configurations among 9910 distinct shift configurations in the 95% credibility set estimated from topC-1; **b**: Plots of the 9 most frequent shift configurations among 12454 distinct shift configurations in the 95% credibility set estimated from topA-0. Rate shifts are indicated on respective branches as black circles; f=frequency in the credible shift set. Source data for this figure can be found in the Dryad repository at https://doi.org/10.5061/dryad.08kprr54m (folder 3.2)[86].

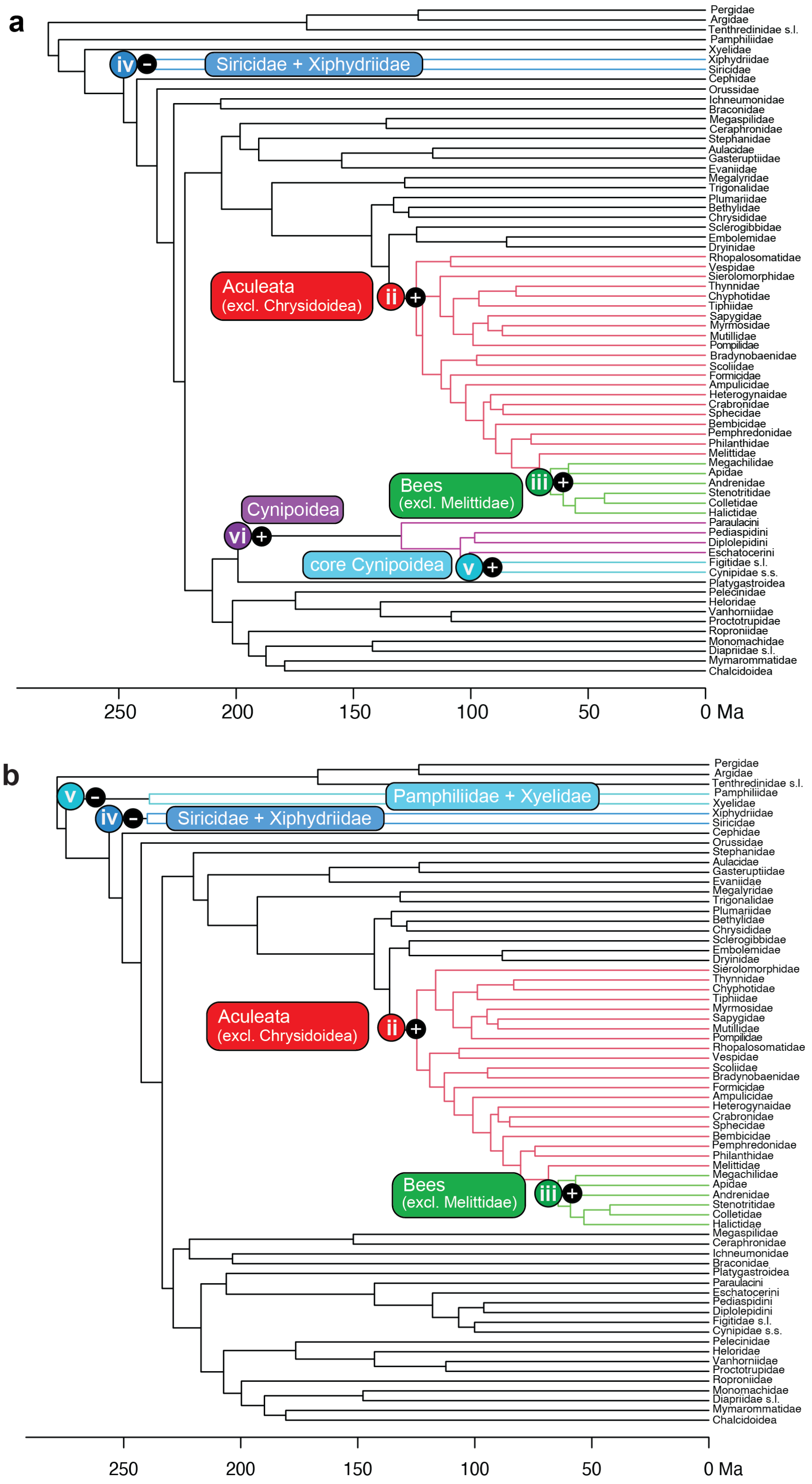

Supplementary Figure 22: Diversification rate shifts estimated with step-wise AIC. a: Diversification rate shifts estimated with MEDUSA based on topology C-1. **b:** Diversification rate shifts estimated with MEDUSA from topology A-0. "+" denotes an increase in rate, "-" = decrease in rate. For rates and other information on specific shifts, see Table 3 in the main text. Source data for this figure can be found in the Dryad repository at https://doi.org/10.5061/dryad.08kprr54m (folder 3.4)[86].

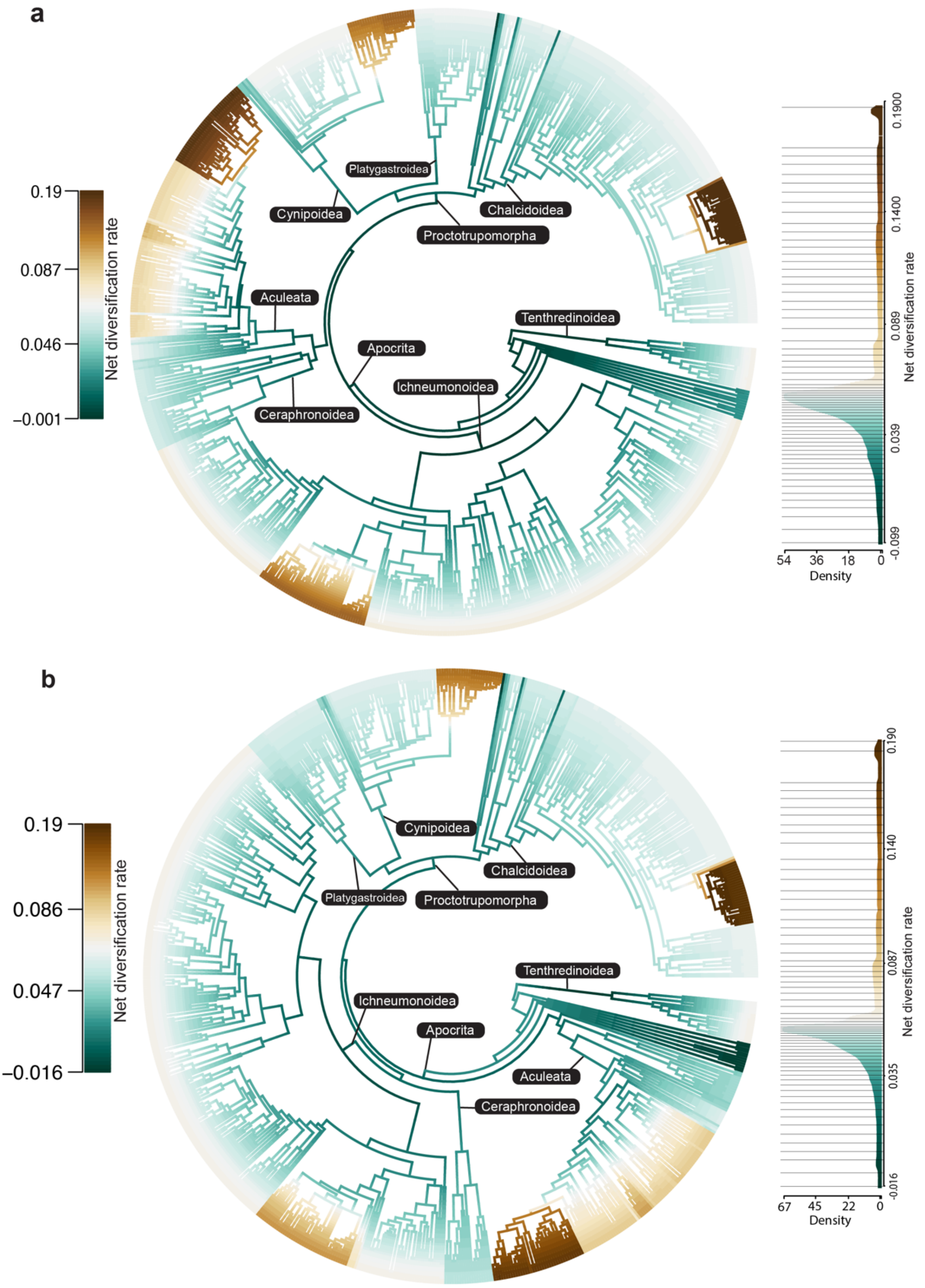

Supplementary Figure 23: Additional diversification results estimated with BAMM. Mean phylorate plots estimated with BAMM, showing mean net diversification rate across the Hymenoptera phylogeny. Histograms further show the frequency of net diversification rate categories in the dataset. **a:** topology C-1. **b:** topology A-0. Source data for this figure can be found in the Dryad repository at https://doi.org/10.5061/dryad.08kprr54m (folder 3.2)[86].

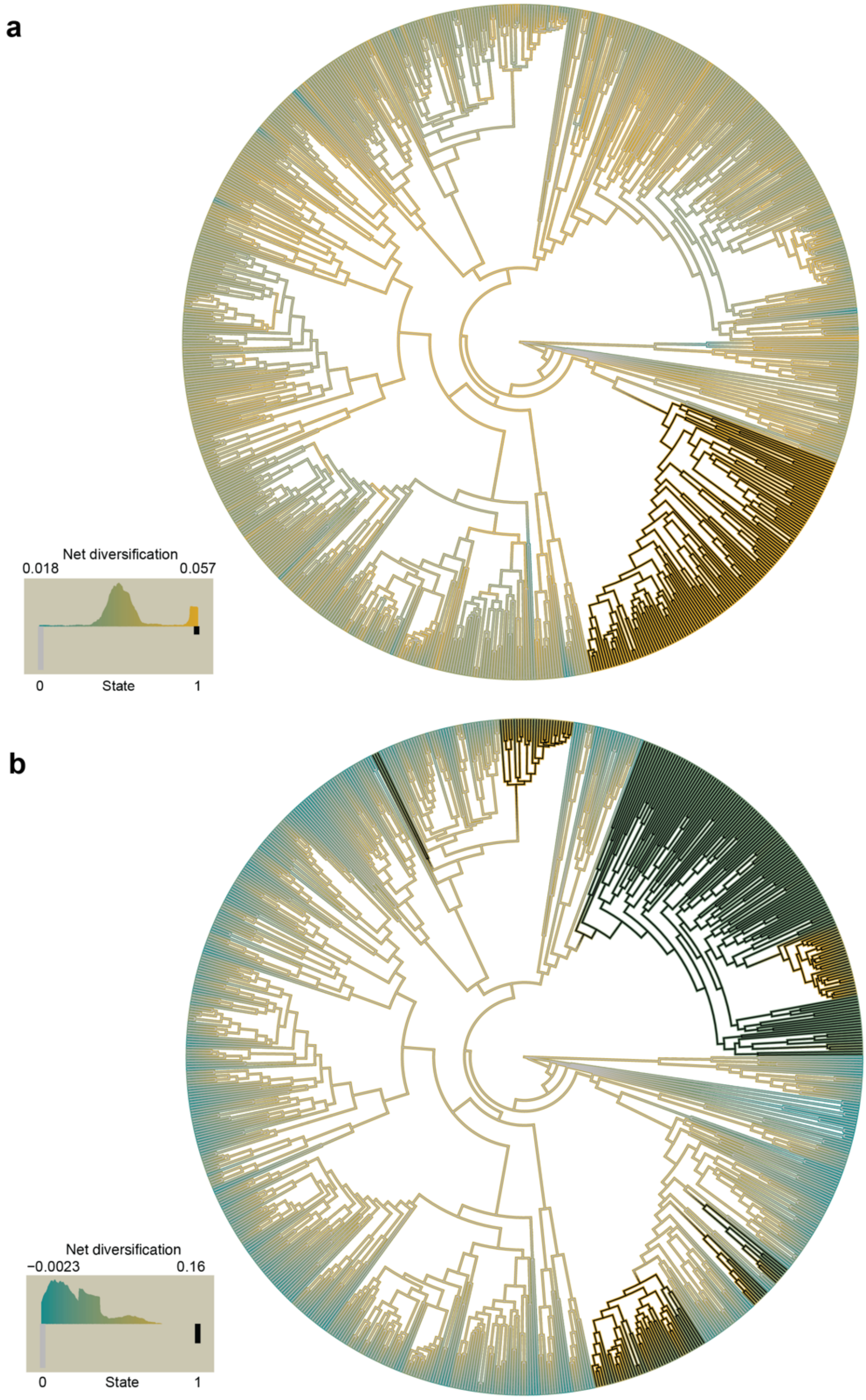

Supplementary Figure 24: **Additional trait-dependent diversification results estimated with HiSSE, part 1. a**: Hidden State Speciation and Extinction (HiSSE) estimate for the presence/absence of the stinger under the best-scoring model. Topology A-0, model: CID-4 - 9 distinct q's. Rates plotted are net diversification rates. **b**: Hidden State Speciation and Extinction (HiSSE) estimate for the presence/absence of secondary phytophagy under the best-scoring model. Topology A-0, model: HiSSE full, irreversible states. Rates plotted are net diversification rates. Source data for this figure can be found in the Dryad repository at https://doi.org/10.5061/dryad.08kprr54m (folder 3.3)[86].

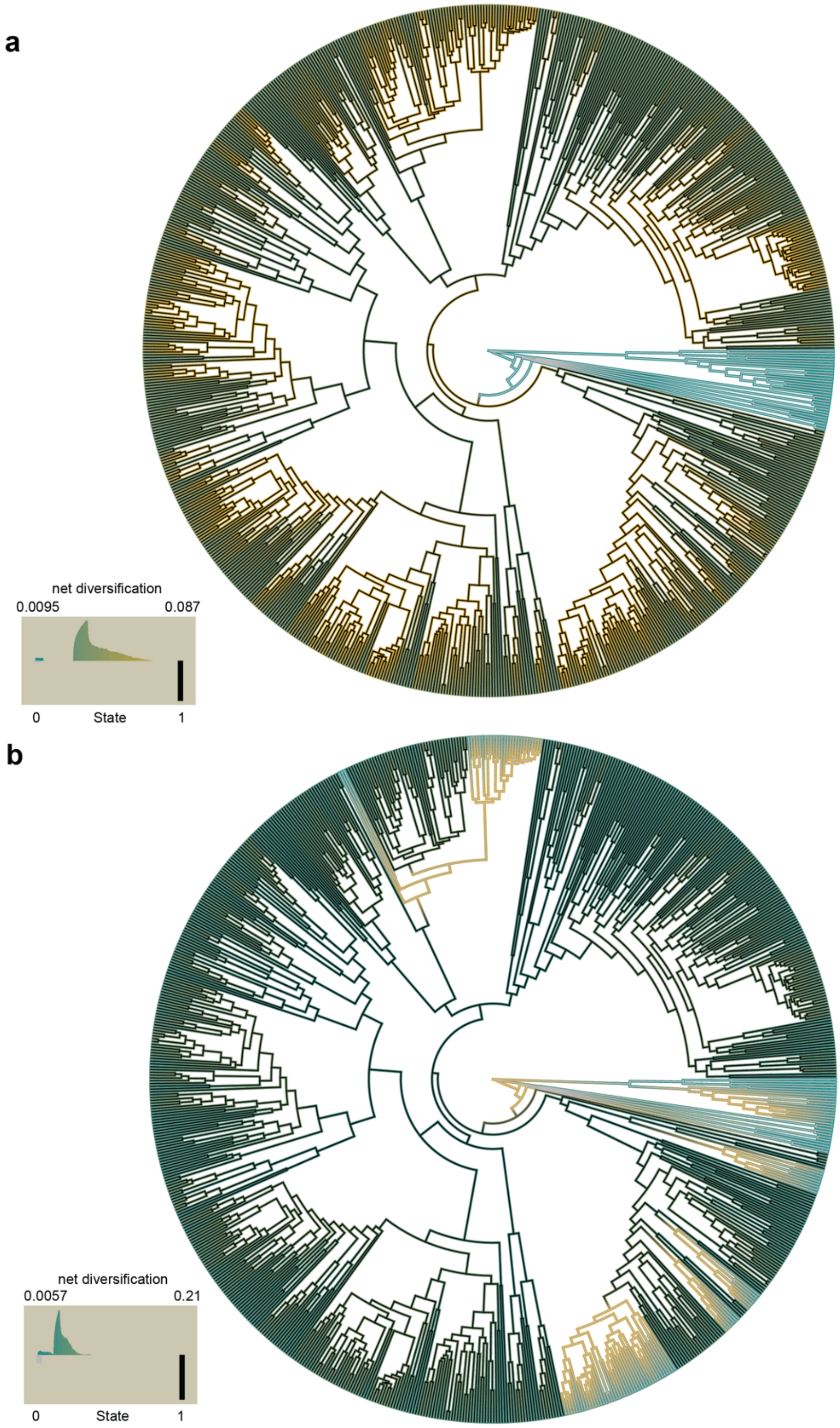

Supplementary Figure 25: **Additional trait-dependent diversification results estimated with HiSSE, part 2. a**: Hidden State Speciation and Extinction (HiSSE) estimate for the presence/absence of the wasp waist under the best-scoring model; topology A-0, model: HiSSE - full, irreversible states. **b**: Hidden State Speciation and Extinction (HiSSE) estimate for the presence/absence of parasitoidism under the best-scoring model; topology A-0, model: HiSSE - all free. Rates plotted are net diversification rates. Source data for this figure can be found in the Dryad repository at https://doi.org/10.5061/dryad.08kprr54m (folder 3.3)[86].

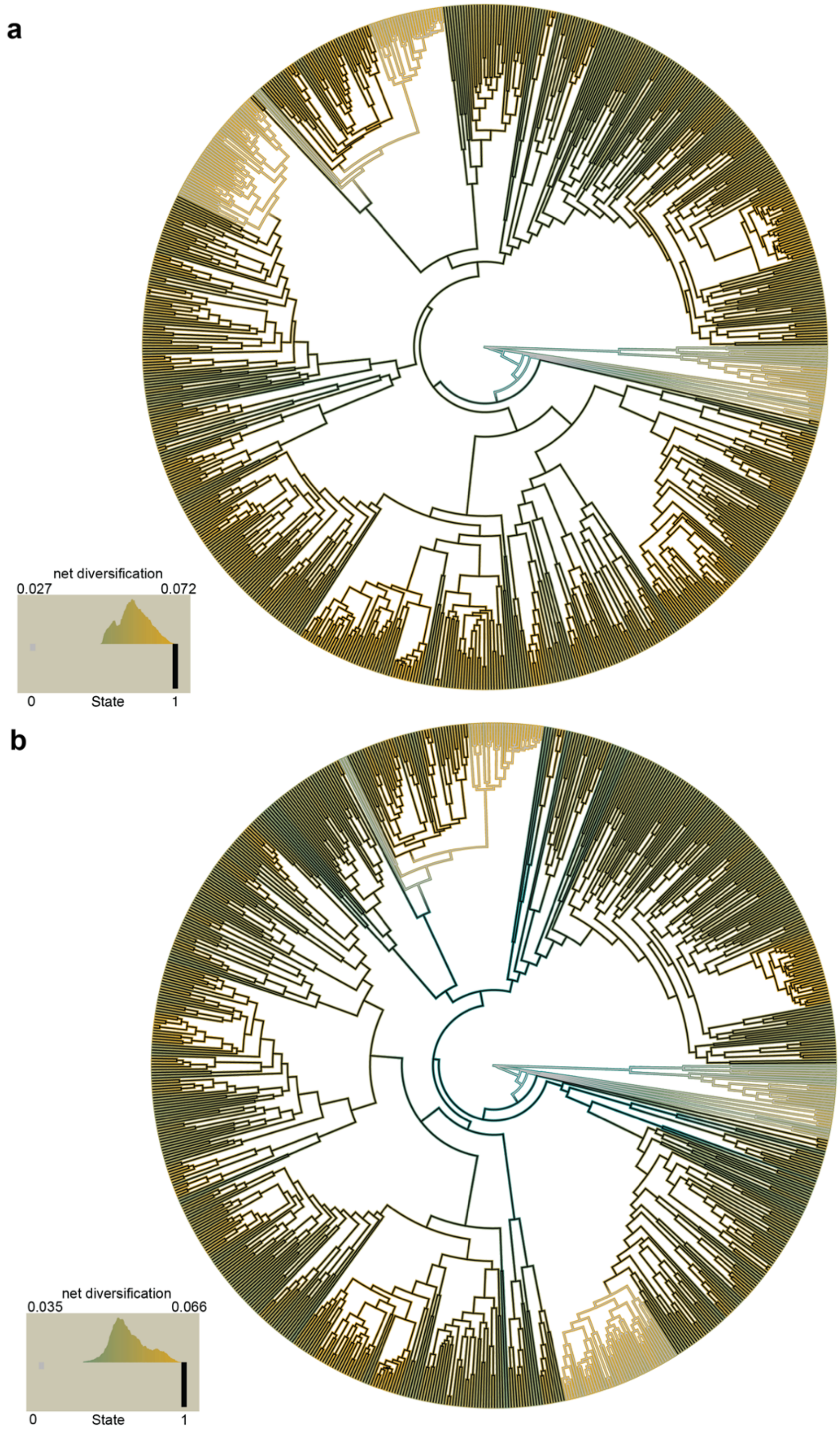

Supplementary Figure 26: Hidden State Speciation and Extinction (HiSSE) estimate for the presence/absence of carnivory under the best-scoring model. **a**: Topology C-1, model: CID-4 - 3 distinct q's. **b**: Topology A-0, model: CID-4 - 3 distinct q's. Rates plotted are net diversification rates. Source data for this figure can be found in the Dryad repository at https://doi.org/10.5061/dryad.08kprr54m (folder 3.3) [86].

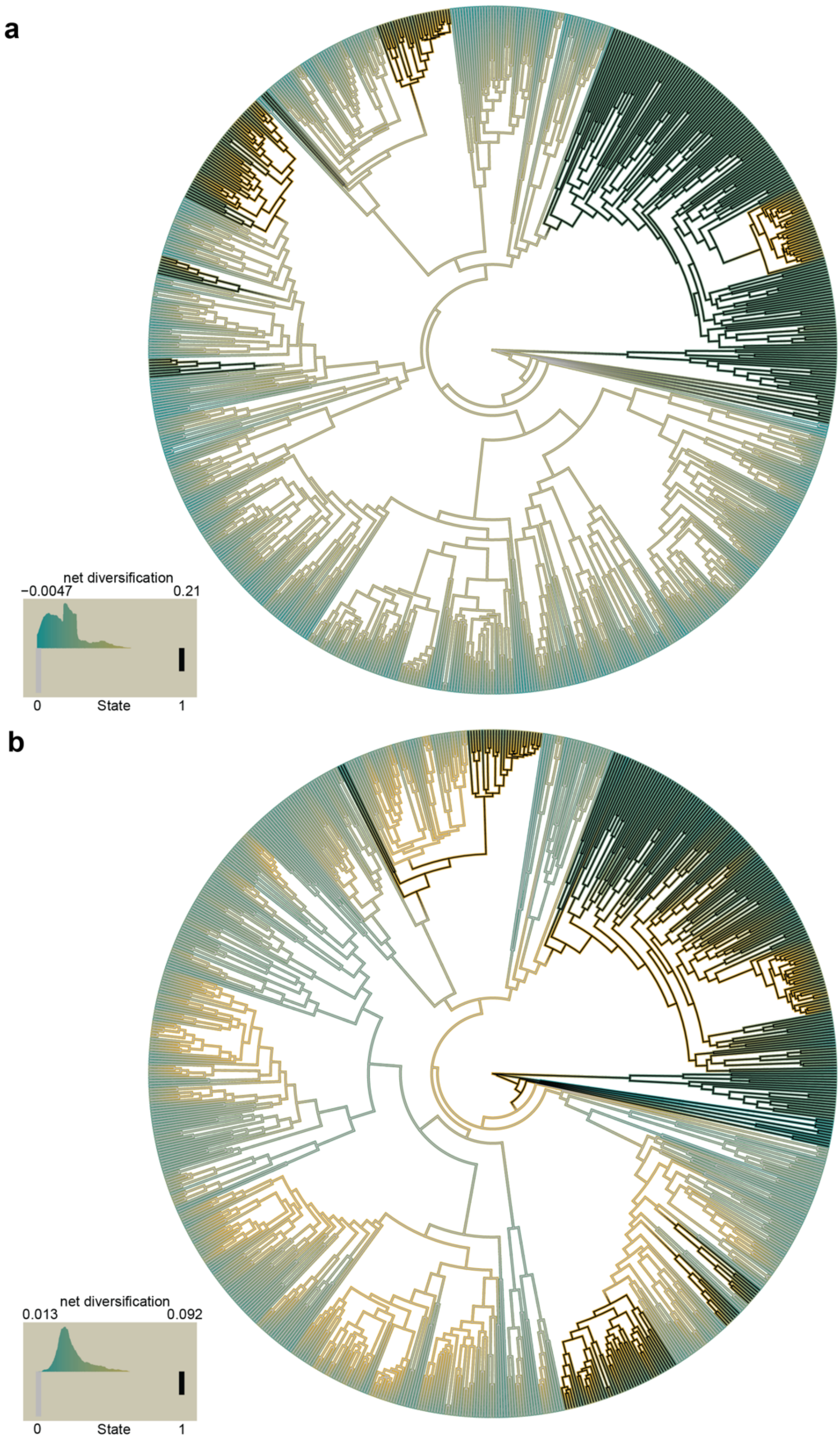

Supplementary Figure 27: Hidden State Speciation and Extinction (HiSSE) estimate for the presence/absence of phytophagy under the best-scoring model. **a**: Topology C-1, model: HiSSE - full, irreversible states. **b**: Topology A-0, model: CID-4 - 9 distinct q's. Rates plotted are net diversification rates. Source data for this figure can be found in the Dryad repository at https://doi.org/10.5061/dryad.08kprr54m (folder 3.3)[86].

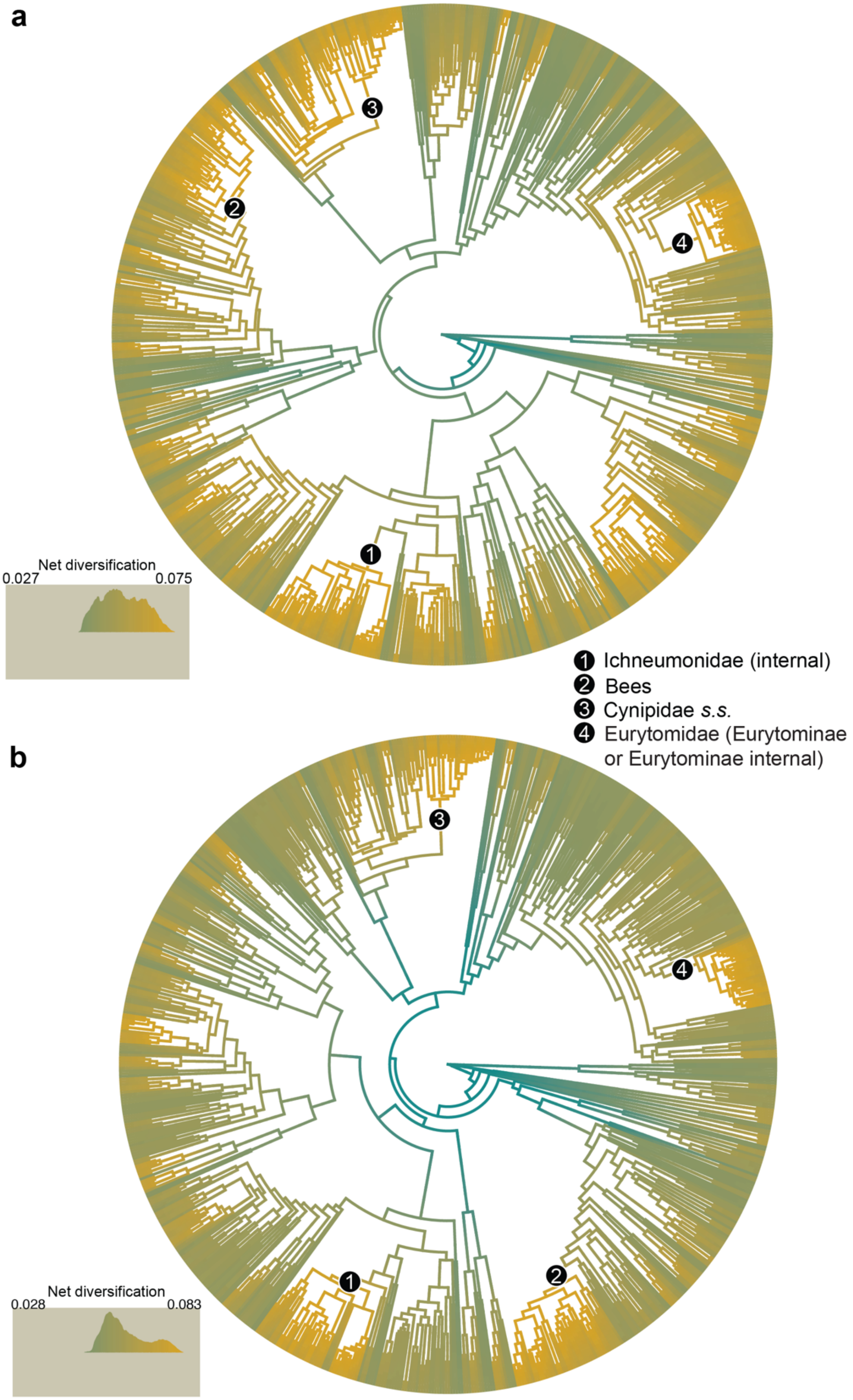

Supplementary Figure 28: Missing State Speciation and Extinction (MiSSE) estimate for the best-scoring model fitted to **a**: Topology C-1 and **b**: Topology A-0. Source data for this figure can be found in the Dryad repository at https://doi.org/10.5061/dryad.08kprr54m (folder 3.5)[86].

Evaluating competing hypotheses on the evolution of Hymenoptera. The most recent phylogenetic hypotheses brought forth on the early evolution of Hymenoptera were using genomic-scale data sets of UCEs[1] and transcriptomes[50], but several other substantial attempts at a comprehensive phylogeny have been made within the last decade or so[51,52,53,54,55,56]. Five of these previous hypotheses are summarized for comparison with our results in Supplementary Fig. 29; we highlight here the best-resolved and most comprehensive results, while for brevity's sake omitting several other important contributions. Many of our phylogenetic results corroborate the findings by some or all of these studies, while others underline where controversies persist.

Proctotrupomorpha: All our analyses confirmed strong support for the Proctotrupomorpha, a clade including the superfamilies Chalcidoidea, Mymarommatoidea, Diaprioidea, Proctotrupoidea, Cynipoidea and Platygastroidea, that had already been recovered by most previous recent analyses (Supplementary Fig. 29a–e[51,52]). Despite some earlier doubts[57], we suggest that the molecular support for this clade here and in previous studies is overwhelming, and Proctotrupomorpha should be treated similarly to Aculeata as a natural clade in hymenopteran evolution. Superfamily relationships within Proctotrupomorpha have been unstable across past studies with every major analysis basically recovering different relationships (Supplementary Fig. 29a–e) but are mostly congruent and well-supported among our analyses. Our results are most similar to Sharkey et al.[55] (Supplementary Fig. 29b). Emerging with robust support from our nucleotide data sets are Platygastroidea and Cynipoidea as sister groups (Supplementary Fig. 2a–e), also found by Sharkey et al.[55] and Klopfstein et al.[56] (Supplementary Fig. 29b and c). Moreover, these two studies recovered Chalcidoidea and Mymarommatoidea (i.e., *Mymaromma*) as sister taxa, and Diaprioidea as sister to the two, again identical to our results. All of our analyses recovered Proctotrupoidea (*s.s.*, sensu Sharkey[49]) paraphyletic with regard to *Ropronia* (Roproniidae), which is a unique result to our study, but that taxon had not been included in any of the cited prior works.

“Evaniomorpha” and Aculeata: A close relationship of Trigonaloidea and Aculeata has been shown by most prior comprehensive phylogenetic work (Supplementary Fig. 29a,c–e). However, not all analyses had been able to also include Megalyridae. Therefore, our results of a joint Trigonaloidea + Megalyroidea being sister to Aculeata had so far only been recovered by Klopfstein et al.[56] with weak support. Our study lends further support for Trigonaloidea + Megalyroidea, as this result is corroborated in all our main analyses (Supplementary Fig. 2a–f) based on nuclear and amino acid data alike. The name Evaniomorpha was originally coined by Rasnitsyn[58] to include Ceraphronoidea, Evanioidea, Stephanoidea, Trigonaloidea and Megalyroidea. Monophyly was never corroborated; rather, most subsequent studies found “Evaniomorpha” a paraphyletic grade with respect to Aculeata, similar to our results. Relationships of the remainder of the “Evaniomorpha” (Ceraphronoidea, Evanioidea, Stephanoidea) were found unstable across our analyses (Supplementary Fig. 2), as well as in previous studies (Supplementary Fig. 29a–e) and will have to be investigated in the future with more extensive taxon sampling. However, similar to some previous studies (Supplementary Fig. 29a, d–e;[1,50,54]) both Evanioidea and Stephanoidea were always recovered as close relatives to Aculeata, Trigonaloidea and Megalyroidea.

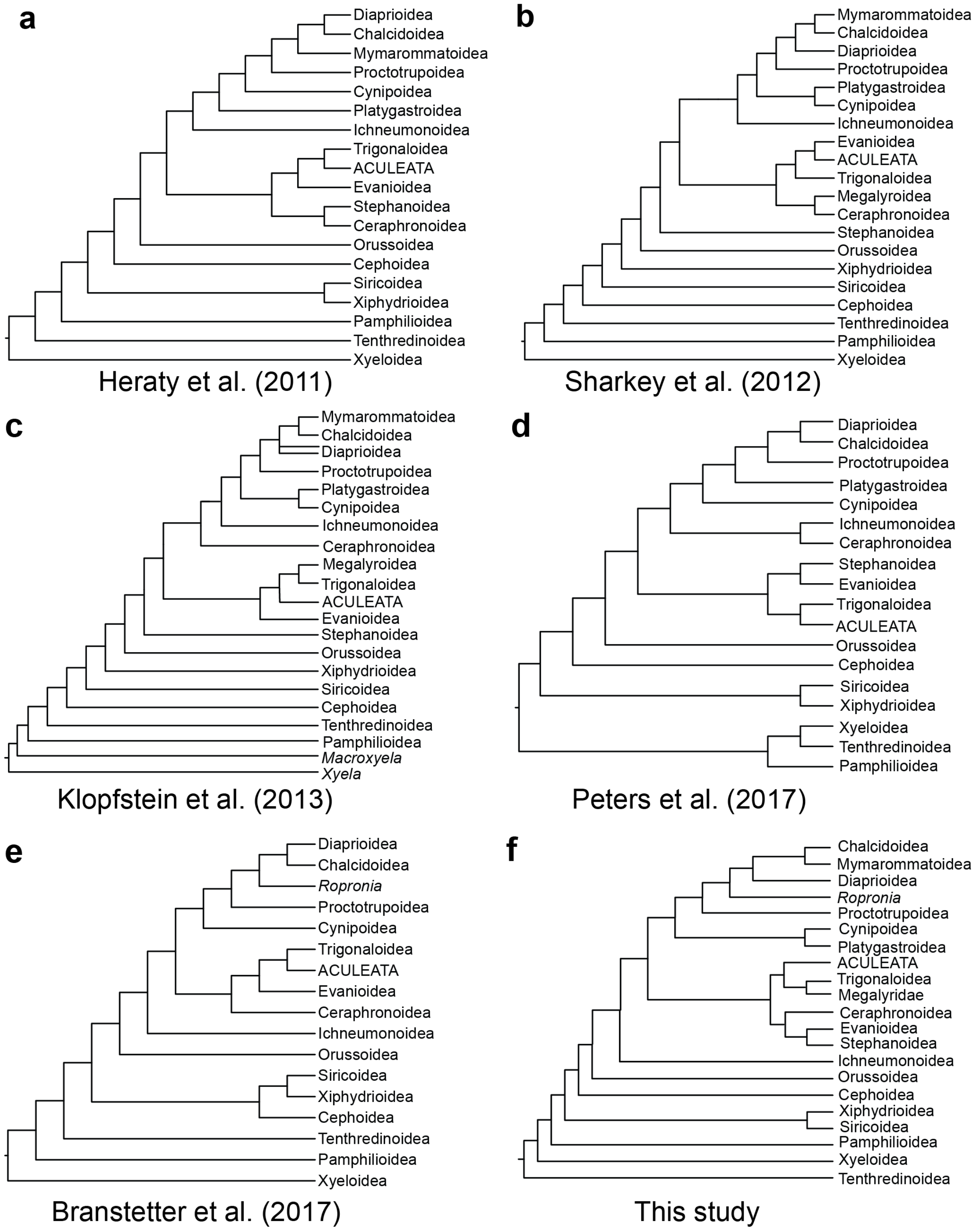

Supplementary Figure 29: Summary of previous phylogenetic hypotheses. Simplified summary trees re-drawn from five major previous analyses of hymenopteran relationships. We cite specific, best-supported results from these studies as often several trees with partially conflicting results were presented. **a**: Heraty et al.[54], maximum likelihood analysis in Fig. 2; **b**: Sharkey et al.[55], simplified total evidence consensus in Fig. 1; **c**: Klopfstein et al.[56], total evidence tree depicted in Fig. 6; **d**: Peters et al.[50], Fig. 1; **e**: Branstetter et al.[1], Fig. 1; **f**: this study, best-supported and favored hypothesis (topC-1).

Sawflies and the sister group to all Hymenoptera: Virtually all previous analyses including non-hymenopteran outgroups have supported Xyeloidea as sister group to the rest of the order[52,54,55,56], a result also corroborated by analyses with a more comprehensive sawfly sampling[59,60,61]. Uniquely, we found Tenthredinoidea recovered as sister group to all remaining Hymenoptera with strong support based on our nucleotide data sets (Supplementary Fig. 2a–e). Peters et al.'s analyses ([50]; Supplementary Fig. 29d) had suggested Eusymphyta (Pamphilioidea (Tenthredinoidea + Xyeloidea)) as sister to the remaining hymenopterans, which is similar to our results from the protein-coding, amino acid translated matrix that recovered (Tenthredinoidea (Xyeloidea + Pamphilioidea)) in this position. All our data sets agree on the relationships of the remaining sawfly lineages to Apocrita as a grade, with Orussoidea being sister to Apocrita and Siricoidea and Xiphydrioidea forming a clade (Supplementary Fig. 2a–f). These results are largely congruent with previous studies, and a sister-group relationship between Siricoidea and Xiphydrioidea was already supported in Heraty et al.[54] and Peters et al.[50]. Our result of the species-rich Tenthredinoidea being sister to the remaining order rather than the species-poor Xyeloidea, a clade that has often been dubbed "primitive", could have tantalizing implications for the evolution of Hymenoptera. Given our generally sparse sampling of sawfly diversity these relationships cannot be regarded as fully resolved, but our results will hopefully re-invigorate the debate and motivate new investigations.

Remaining uncertainties: Despite the magnitude of our data set, our study clearly shows that the early evolution of Hymenoptera presents a difficult phylogenetic problem. Uncertainties remain regarding the position of two lineages: Ichneumonoidea and Ceraphronoidea. Consulting previous analyses (Supplementary Fig. 29a–e), it is clear that these taxa were already unstable and have been highlighted as problematic in analyses[54, 55, 56]. Increasing GC content among UCE loci has been shown to negatively affect phylogenetic reconstruction and cause incongruences[62], but we did not identify such a trend in our data set. The levels of GC content across the different main matrices were relatively similar, albeit slightly higher in the protein-coding subset of loci (0.45–0.46 vs 0.49). The two bins that recovered topology B (Ichneumonoidea sister to Proctotrupomorpha, also recovered by the main protein-coding data analysis), have both low and medium levels of GC content (averages 0.31 and 0.49). The bin with the highest GC content inferred topology C (Ichneumonoidea sister to Apocrita), yet the composition of loci in the nuc-70% matrix (which inferred topology C) has fairly equal contributions from high and low GC bins. By contrast, topology A (Ichneumonoidea sister to Ceraphronoidea), the result recovered from the larger, less complete data matrices (NUC-50% and NUC-60%), is not inferred from the GC bins at all. Peters et al.[50] had first shown a sister-group relationship between Ichneumonoidea and Ceraphronoidea, with the two of them in turn sister to Proctotrupomorpha (Supplementary Fig. 29d), but no conflict in the data or incongruent results are mentioned in that paper[50].

Other properties of the UCE loci may therefore be driving the observed incongruences regarding the placement of Ichneumonoidea and Ceraphronoidea, but one issue prevented us from pinpointing and excluding the exact culprits of conflict in the data. The edge-trimmed locus alignments are very short as much of the variation (presumably most flanking, but also some core regions) is trimmed due to the broad phylogenetic scale of the taxon set. This leaves individual loci that are, for example, not suitable for gene tree reconstruction to extract phylogenetic information on the position of the two problem taxa. This issue also necessitated assigning large bin sizes for GC filtering to reconstruct resolved phylogenies. We had initially tried to reduce the stringency of alignment trimming in order to

recover longer UCE alignments (see above); however, relationships inferred from these relaxed alignments were simply not plausible.

Given this impasse regarding a potential exclusion of loci that could be biasing phylogenetic inference one way or the other, we attempted to evaluate support for each of the three placements of Ichneumonoidea and the three positions of Ceraphronoidea (Supplementary Table 1) with each main data set by breaking these down to four hypotheses that could be addressed using FcLM (Supplementary Data 5 and 14). Information content in all alignments was high, as demonstrated by high numbers of decisive quartets, but the support for placement of Ichneumonoidea in position A (sister to Ceraphronoidea, both sister to Proctotrupomorpha) or position B (sister to Proctotrupomorpha) is basically equivocal. The best-supported position of Ichneumonoidea is C (sister to Apocrita), receiving 36.5–43.5% support of the decisive quartets – obviously not overwhelming support but the best choice among the inferred options. Ceraphronoidea's position within the larger Evaniomorpha-Aculeata clade was further evaluated in the context of two hypotheses (Supplementary Data 5), which rendered highest support for Ceraphronoidea being sister to Evanioidea (44.5–57.2%) or Evanioidea + Stephanoidea (42.8–50.3%) by the nucleotide matrices. A sister group relationship with Stephanoidea received little decisive quartets (11.1–11.9%). Interestingly, a sister group relationship of Ceraphronoidea with Evanioidea was not supported by any of our phylogenetic analyses, but it could be that the support of Ceraphronoidea's position shifts once Ichneumonoidea is excluded as a potential confounding factor. Based on FcLM support, we conclude that a sister group relationship of Ichneumonoidea with Apocrita (topology C) is the most likely, while a grouping of Ceraphronoidea with Evanioidea + Stephanoidea (or potentially Evanioidea only) is our current best estimate. These relationships are recovered in our topology C-1 (Fig. 1 and Supplementary Fig. 2a), estimated from partitioned analyses of the most complete 70% UCE alignment, which we consider our best hypothesis regarding superfamily relationships in Hymenoptera. We have used this result and tree for macroevolutionary analyses in comparison with topology A-0 (Supplementary Fig. 2c–e), as this result was recovered by Peters et al.[50] using transcriptomes and still remains a contending hypothesis.

We reviewed morphological evidence for the alternative positions (A,B,C; Supplementary Fig. 2a–f) of Ichneumonoidea and Ceraphronoidea, but were not able to find much support for either hypothesis based on previously described characters. Shared characters between Ceraphronoidea and Ichneumonoidea are paired translucent patches on the metasoma (also shared with Orussidae[63]) and the presence of a uniquely structured ovipositor moving muscle (S7-first valvifer[64,65,66]) that has not been recorded from other taxa and might be involved in the activation (protraction) of the ovipositor assembly. Another potential synapomorphy between Ichneumonoidea and Ceraphronoidea could be the presence of a single vein along the anterior wing margin[67,68]. In both cases, it appears this feature is based on a fusion of the C+R (Costa and Radius), although it is difficult to homologize wing venation in these two groups due to reduced wing venation. Further, both Ichneumonoidea and Ceraphronoidea share a distinctly developed lateral metacoxal articulation with Evanioidea, Stephanoidea and Megalyroidea[52]. Sharkey et al.[55] quote ten morphological synapomorphies for a grouping of Ichneumonoidea as sister to Proctotrupomorpha, for example, the occipital carina reaching the hypostome ventrally and the teeth on left mandible reduced to three[55]. Not all these conditions occur in all ichneumonoids, and this position was the least supported by our molecular data. Lastly, we were unable to identify characters to support a relationship of Ichneumonoidea as sister to

all Apocrita (topC-1, best supported by our molecular data). Support for such a grouping would necessarily be (even more) difficult to find given the age of the clade and the multitudes of secondary morphological modifications that apocritan groups have undergone since their most recent common ancestor. Closer investigations of internal anatomy may shed more light on putative morphological synapomorphies in the future.

Overall, some uncertainties remain in the phylogenetic relationships among hymenopteran superfamilies even with the application of genomic-scale datasets in this and previous studies. Whether these persisting issues will be resolved with the addition of more molecular data is uncertain. For the most part, however, we were able to show that the different phylogenetic placements of Ichneumonoidea and Ceraphronoidea do not affect our conclusions on ancestral reconstructions or diversification estimates.

Comparison of divergence dating results. We estimated divergence ages using MCMCTREE and 12 fossil calibrations based on two different data sets and topologies (the nuc-50% matrix with topA-0 and the nuc-70% matrix with topC-1). Our results show little deviation between the two analyses (0.2–15.9 Ma, average 3.9 Ma), and most differences can be attributed to differences in topologies. However, our results differ to some degree from the age estimates recovered in other recent dating analyses on Hymenoptera (Supplementary Data 6). For example, Branstetter et al.[1], Peters et al.[50] and Ronquist et al.[69] estimated the age of Vespina (and therefore the origin of parasitoidism) with ~200 Ma, ~247 Ma and ~270 Ma, respectively. Our estimates for Vespina range between 233–247 Ma, and thus are much older than Branstetter et al.'s[1], slightly younger than Peters et al.'s[50], and much younger than Ronquist et al.'s[69] estimate. In general, the estimates by Branstetter et al.[1] are younger than ours, while Ronquist et al.'s[69] estimates are older (Supplementary Data 6). In comparison to Peters et al.[50], there is no clear trend but it appears that earlier evolutionary divergence events (e.g., for Unicalcarida, Vespina, Apocrita) are estimated as somewhat younger, while crown ages for parasitoid clades are estimated older in our study (e.g. Ichneumonoidea, Ceraphronoidea) than in theirs[50]. Reasons for these incongruities could be multifold. For one, they are significant differences in taxon sampling between ours and the previously mentioned analyses. The Branstetter et al.[1] and Peters et al.[50] data sets were rich in aculeate taxa, whereas non-aculeate lineages were not well sampled. Ronquist et al.[69] on the other hand focused on the early evolutionary history of Hymenoptera and included extensive sampling of sawfly and woodwasp lineages. Due to this more expansive taxon sampling, we would expect their dating analyses to provide more robust results in this part of the Hymenoptera tree. For the same reason, we assume our results to be more robust concerning dating of apocritan lineages than these three previous studies.

However, it is difficult to assess the effect of taxon sampling within this comparison of divergence ages since other aspects of the analyses were not equal. All analyses used different sets of fossil calibrations. Peters et al.[50] used 14 fossil calibrations, a similar number to our 12, but only four fossils overlap between theirs and our analyses. Branstetter et al.[1] used 37 fossils, many of them nested within each other and placed on more recent nodes. Ronquist et al.[69] employed 45 fossils in a tip-dating or total-evidence approach (vs node-dating in ours and the other two studies) which generally has been found to recover older divergence ages[70,71]. The type of the sequence data analyzed also differed between these analyses: only Branstetter et al.[1] used UCEs like us, whereas Peters et al.'s [50] analysis was based on transcriptomes, and Ronquist et al.[69] used a comparatively small data set of seven mitochondrial and nuclear markers. When comparing divergence ages estimated from UCEs to those estimated from smaller Sanger sequencing nuclear datasets,

Blaimer et al. [72] found that estimates based on UCEs were slightly younger, for example. Lastly, different methods of divergence dating have been employed across these studies and may have had an impact on the results. Similarly to us, Peters et al.[50] have used MCMCTREE, but Branstetter et al.[1] used BEAST[73], whereas Ronquist et al.[69] employed MrBayes 3.2[74]. Given all the above variables, it seems premature to declare one set of estimates as superior to the others *per se*, however, we do consider our estimates as major improvements for Apocrita given our expanded, more balanced sampling.

It is also understood that the discovery of new fossil evidence could have an unforeseeable impact on our current estimates (as well as previously published ones) and the diversification patterns derived from them. Fossils that improve knowledge of divergence timing within Hymenoptera could also prove crucial to better link innovations with diversification events and to some degree alter the conclusions drawn in our study.

Methodological considerations for macroevolutionary analyses.

Character evolution: An obvious limitation of our ancestral character reconstructions of hymenopteran life strategies was that character states were coded on a family basis or in some cases, such as Chalcidoidea or Platygastroidea, on a superfamily basis. Ancestral states for families were assigned by integrating over a diversity of life strategies in each clade, using a “majority rule” approach that necessarily neglected isolated secondary species-level transitions from parasitoidism to phytophagy or vice versa. We chose this approach to implement the most complete view of character diversity in each group by accounting for unsampled taxa and avoiding any bias by over- or underrepresentation of particular states. Representing each terminal diligently by the species’ actual state (if known) would most likely not be fully representative of the diversity of behaviors in the respective families or clades and could lead to incorrect ancestral estimations. For the most rigorous analysis, one would estimate ancestral states for larger clades from terminals in next-to-complete genus or even species-level phylogenies, and then use these estimates as input in a large-scale framework analysis such as ours. For Hymenoptera, this is hardly feasible at this point in time, as we are lacking both the detailed phylogenies to estimate family-level ancestral states and, most importantly, the natural history data for a majority of the species. Our approach to coding character states was therefore a necessary approximation to estimating the evolution of life strategies in Hymenoptera. We do believe that coding families based on this majority rule is likely to assign states to families that in most cases adequately represent the ancestral states of the group (unless there was a switch to a different strategy early in the evolution of the group) and lead to a reasonably accurate picture of the evolutionary history of life strategies in Hymenoptera.

For Chalcidoidea, we had to apply an even coarser approach as several families within chalcidoids are not monophyletic and coding on a lineage specific basis (similar to Cynipoidea) was not possible because of the lack of natural history information for a good part of the taxa. We further coded Chalcidoidea as polymorphic including both parasitoid and secondarily phytophagous species (rather than applying majority rule), because this group shows a particular diversity of life histories paired with a high level of uncertain states. Given the position of Chalcidoidea within Proctotrupomorpha (all parasitoids), it is unlikely, however, that this conservative coding has had much influence on ancestral reconstructions elsewhere in the phylogeny.

A general concern with ancestral reconstructions performed on incomplete phylogenies is that changes in the level of detail of the analyses and associated character coding could result in different estimated rates of character evolution and conclusions. In our

case, coding on a clade-level basis may have homogenized the rates of character evolution estimated across our phylogeny, potentially leading to the ER model being recovered as best-fitting. An analysis on species-level trait data or coding on a different taxonomic level may result in the ARD model to perform better, affecting the inferred rates of character evolution and possibly the reconstructed states. We therefore compared reconstructions under the ARD model with those from the ER model, and confirmed that the reconstructions under the two different models are essentially the same, indicating that our conclusions on trait evolution are robust to changes in estimated transition rates. As outlined above, the clade-level coding was chosen as the current best approach to represent the entire character state diversity in each clade and to avoid bias by over- or under-representation of states. We argue that this approach is appropriate for the goal to highlight broad evolutionary patterns in Hymenoptera by focusing on shifts in the early diversification of the order, although it presents a necessary approximation to a species-level approach.

Lastly, we realize that our coding scheme may very well represent an oversimplification of diverse biological traits, ignoring for example endo- and ectoparasitism, koino- and idiobiosis, and egg/larval/pupal stage parasitoids. Initially, these traits were coded as such across the matrix, but we soon rejected this approach as much of the matrix would be coded as uncertain given many of the included taxa lack host data. Further, inquilines are particularly difficult to code within this scheme, as they are arguably either secondary phytophages, parasitoids or possibly predators. Inquilinism, along with hyperparasitoidism may be more commonplace than currently known; working out precisely who is parasitizing or feeding on whom is not trivial, especially in gall systems. Collectively, our approach to coding at the level of "parasitoid" is therefore the most conservative means of addressing the question of when parasitoidism arose in evolutionary time.

Diversification rate analyses: Our approach to estimate diversification rates in Hymenoptera was to integrate over several different methodologies, namely BAMM[30, 31], MEDUSA[41], and HiSSE[43]. While the first two methods allow only for an estimation of diversification rate shifts that then *a posteriori* can be associated with potential key innovations, HiSSE allows for an explicit test if a trait is associated with the diversification dynamics of a phylogeny, but does not estimate rate shifts per se. A reassuring result was that all methods agreed on non-melittid bees to share an increased diversification rate regime, with a higher net diversification rate estimated than for any other lineage in Hymenoptera. However, the three methods otherwise showed variation regarding which clades were identified with an ancestral rate increase or decrease. This begs the question which method inferred the best estimate and if there are methodological considerations that could help in deciding this.

BAMM is a widely used method that allows for incomplete sampling by assigning both a global sampling fraction across the entire phylogenetic tree and clade-specific sampling fractions to compensate for incomplete sampling within clades. However, it is unclear up to what level of incomplete taxon sampling these corrections are effective since our phylogenies were clearly extremely incomplete. For very incomplete phylogenies, the BAMM developers recommend placing missing species in the phylogeny using, e.g., PASTIS[75], but for a data set of our magnitude (>150,000 described species) this approach was not feasible. Chang et al.[76] have also suggested that the sampling fraction method to account for incomplete sampling in diversification analyses, as implemented in our BAMM analysis, may have low power to estimate diversification models in very incomplete phylogenies. A taxonomic method, in which clades are collapsed to terminal lineages of equal rank, may be more appropriate for the latter[76], which is the reason why we estimated

diversification rates and rate shifts from our data set also using the stepwise AIC method in MEDUSA. However, stepwise AIC methods are limited in that they estimate a single best rate shift model and configuration, while there may be many combinations of shifts that have very similar probabilities.

BAMM has been criticized as being very sensitive to the selected rate shift prior[77]. These criticisms have since been invalidated by the developers[78], but more concerns were raised regarding the underestimation of rate shifts[79] and overestimation of diversification rates by BAMM[80]. Thus, the use of other approaches, such as stepwise AIC, helped alleviate some of the concerns raised in the recent debates about BAMM by providing additional or alternative support. For one, two shifts were supported by both BAMM and MEDUSA (non-chrysidoid aculeates and non-melittid bees), and we found no indication of an overestimation of diversification rates by BAMM in these clades. In fact, the net diversification rates estimated by both methods were remarkably similar (non-chrysidoid aculeates: 0.081 vs 0.085–0.091; non-melittid bees: 0.151–0.151 vs 0.154–0.156).

A further limitation of MEDUSA analyses was that they required collapsing the phylogeny to a mostly family-level tree (as for ancestral character reconstructions), which necessarily estimates a coarser picture of diversification dynamics than the sampling fraction approach implemented by BAMM. The reason why several strong rate shifts that were suggested by BAMM could not be confirmed with the stepwise AIC method in MEDUSA is simple: since the phylogeny was collapsed to the family-level, the method only allows to detect rate shifts *above* the family level. There was no possibility to detect the rate shifts for Cynipidae *s.s.*, Eurytomidae (Eurytominae) and Ichneumonidae with MEDUSA. Because of the two dually supported rate shifts and this obvious limitation, we are inclined to also believe in the shifts supported exclusively by BAMM, at least when these were supported by analyses of both topA-0 and topC-1 trees.

It is further possible that our clade-level approach to character coding could have resulted in a similar bias of shifts occurring prior to or at the base of clades for BAMM, HiSSE and MEDUSA analyses. This is more likely for the Medusa estimates as these were performed on a clade-level tree only and necessarily limited to shifts prior to the base of these clades. HiSSE recovered a rate increase in Eurytomidae, a clade that was sunk for the purpose of the analyses within the larger Chalcidoidea clade. This result that was also confirmed by BAMM analyses performed on a species-level tree while excluding all trait information. BAMM analyses also recovered a shift within Ichneumonidae, giving further notion to believe that the placement of shifts in these analyses was not influenced by our clade-level character coding. Nonetheless, the possibility of such a bias exists.

Trait-dependent analyses: If one has prior assumptions as to which traits may be causing an acceleration (or slow-down) of diversification rate, as in our case, perhaps a more elegant method is the HiSSE framework, because it allows for an *a priori* association of traits with the diversification dynamics of a phylogeny. The HiSSE framework has been developed explicitly to be less susceptible to type I error (i.e., inferring presence of trait-dependency incorrectly) than its predecessor BiSSE (Binary State Speciation and Extinction[81]), which is a benefit of the more complex null models applied in this approach[43,82]. Although HiSSE models have been used in numerous publications over the last years, their performance concerning minimum sample sizes to obtain accurate estimates and the effect of incomplete or unbalanced taxon sampling remains unclear. The latter information can be incorporated using state-specific sampling probabilities, which we applied for our analyses. The BAMM developers have cautioned against overly relying on support for trait-dependent

diversification for traits that have evolved only once or few times[47], which is basically the case for all our key innovations, except perhaps for secondary phytophagy. Since the three rate shifts for Cynipidae *s.s.*, Eurytomidae (Eurytominae) and non-melittid bees estimated in the trait-dependent model for secondary phytophagy were estimated by BAMM as well, we place confidence in these shifts. However, while our taxon sampling for Cynipoidea and bees was balanced, Chalcidoidea was a much more challenging group in terms of lineage sampling, and we cannot discount that a possible bias could drive the accelerated diversification rates seen in Eurytominae. The support for trait-dependent diversification of the wasp waist (as key innovation in Apocrita) and parasitoidism (as key innovation in Vespina) remained somewhat equivocal as there were only small rate increases estimated for these clades by BAMM, with no shifts in the 95% credibility set. In other studies, where both BAMM and HiSSE models have been used side by side, diversification rates and estimated shifts often are broadly consistent[47,83,84], but can also be quite divergent[85].

Supplementary References

1. Branstetter, M.G. et al. Phylogenomic insights into the evolution of stinging wasps and the origins of ants and bees. *Curr. Biol.* **27**, 1019–1025 (2017).

2. Blaimer, B.B., Ward P.S., Schultz T.R., Fisher B.L. & Brady S.G. Paleotropical diversification dominates the evolution of the hyperdiverse ant tribe Crematogastrini (Hymenoptera: Formicidae). *Insect Syst. Div.* **2**, 3 (2018).

3. Faircloth, B.C., Branstetter M.G., White N.D. & Brady S.G. Target enrichment of ultraconserved elements from arthropods provides a genomic perspective on relationships among Hymenoptera. *Mol. Ecol. Res.* **15**, 489–501 (2015).

4. Cruaud, A. et al. Optimized DNA extraction and library preparation for minute arthropods: application to target enrichment in chalcid wasps used for biocontrol. *Mol. Ecol. Res.* **19**, 702–710 (2019).

5. Cruaud, A. et al. Ultra-Conserved Elements and morphology reciprocally illuminate conflicting phylogenetic hypotheses in Chalcididae (Hymenoptera, Chalcidoidea). *Cladistics* **37**, 1–35 (2021).

6. Rasplus, J.-Y. et al. A first phylogenomic hypothesis for Eulophidae (Hymenoptera, Chalcidoidea). *J. Nat. Hist.* **54**, 597–609 (2020).

7. Blaimer, B.B., Gotzek D., Brady S.G. & Buffington M.L. Comprehensive phylogenomic analyses re-write the evolution of parasitism within cynipoid wasps. *BMC Evol. Biol.* **20**, 155 (2020).

8. Santos, B.F., Perrard A. & Brady S.G. Running in circles in phylomorphospace: host environment constrains morphological diversification in parasitic wasps. *Proc. R. Soc. B: Biol. Sci.* **286**, 20182352 (2019).

9. Santos, B.F. et al. Phylogenomics of Ichneumoninae (Hymenoptera, Ichneumonidae) reveals pervasive morphological convergence and the shortcomings of previous classifications. *Syst. Entomol.* **46**, 704–724 (2021).

10. Supeleto, F.A., Santos B.F., Brady S.G. & Aguiar A.P. Phylogenomic analyses reveal a rare new genus of wasp (Hymenoptera, Ichneumonidae, Cryptinae) from the Brazilian Atlantic Forest. *System. Biodivers.* **18**, 646–661 (2020).

11. Jasso-Martinez, J.M. et al. Phylogenomics of braconid wasps (Hymenoptera, Braconidae) sheds light on classification and the evolution of parasitoid life history traits. *Mol. Phylogenet. Evol.* **173,** 107452 (2022).

12. Tagliacollo, V.A. & Lanfear R. Estimating improved partitioning schemes for UltraConserved Elements (UCEs). *Mol. Biol. Evol.* **37**, 1798–1811 (2018).

13. Lanfear, R., Frandsen P.B., Wright A.M., Senfeld T. & Calcott B. PartitionFinder 2: New methods for selecting partitioned models of evolution for molecular and morphological phylogenetic analyses. *Mol. Biol. Evol.* **34**, 772–773 (2017).

14. Lanfear, R., Calcott B., Kainer D., Mayer C. & Stamatakis A. Selecting optimal partitioning schemes for phylogenomic datasets. *BMC Evol. Biol.* **14**, 82 (2014).

15. Nguyen, L.-T., Schmidt H.A., von Haeseler A. & Minh B.Q. IQ-TREE: a fast and effective stochastic algorithm for estimating maximum-likelihood phylogenies. *Mol. Biol. Evol.* **32**, 268-274 (2015).

16. Borowiec, M.L. AMAS: a fast tool for alignment manipulation and computing of summary statistics. *PeerJ* **4**, e1660 (2016).

17. Xie, W., Lewis P.O., Fan Y., Kuo L. & Chen M.-H. Improving Marginal Likelihood Estimation for Bayesian Phylogenetic Model Selection. *Syst. Biol.* **60**, 150–160 (2011).

18. Edgar, R.C. MUSCLE: multiple sequence alignment with high accuracy and high throughput. *Nucleic Acids Res.* **32**, 1792–1797 (2004).

19. Portik, D.M. & Wiens J.J. Do Alignment and Trimming Methods Matter for Phylogenomic (UCE) Analyses? *Syst. Biol.* **70**, 440–462 (2021).

20. Borowiec, M.L. Convergent evolution of the army ant syndrome and congruence in big-data phylogenetics. *Syst. Biol.* **68**, 642–656 (2019).

21. Schoch, C.L. et al. NCBI Taxonomy: a comprehensive update on curation, resources and tools. *Database (Oxford)* (2020).

22. Katoh, K., Asimenos G. & Toh H. Multiple alignment of DNA sequences with MAFFT. In: *Bioinformatics for DNA sequence analysis*). Springer (2009).

23. Castresana, J. Selection of conserved blocks from multiple alignments for their use in phylogenetic analysis. *Mol. Biol. Evol.* **17**, 540–552 (2000).

24. Strimmer, K. & Von Haeseler A. Likelihood-mapping: a simple method to visualize phylogenetic content of a sequence alignment. *Proc. Natl. Acad. Sci. U. S. A.* **94**, 6815-6819 (1997).

25. Mello, B. & Schrago C.G. Assignment of calibration information to deeper phylogenetic nodes is more effective in obtaining precise and accurate divergence time estimates. *Evol. Bioinformat. Online* **10**, 79–85 (2014).

26. Misof, B. et al. Phylogenomics resolves the timing and pattern of insect evolution. *Science* **346**, 763–767 (2014).

27. Wolfe, J.M., Daley A.C., Legg D.A. & Edgecombe G.D. Fossil calibrations for the arthropod Tree of Life. *Earth Sci. Rev.* **160**, 43–110 (2016).

28. Yang, Z. PAML 4: Phylogenetic analysis by maximum likelihood. *Mol. Biol. Evol.* **24**, 1586–1591 (2007).

29. Rambaut, A., Drummond A.J., Xie D., Baele G. & Suchard M.A. Posterior summarization in Bayesian phylogenetics using Tracer 1.7. *Syst. Biol.* **67**, 901–904 (2018).

30. Rabosky, D.L. et al. Rates of speciation and morphological evolution are correlated across the largest vertebrate radiation. *Nat. commun.* **4**, 1–8 (2013).

31. Rabosky, D.L. Automatic detection of key innovations, rate shifts, and diversity-dependence on phylogenetic trees. *PLOS ONE* **9**, e89543 (2014).

32. Rabosky, D.L. et al. BAMMtools: an R package for the analysis of evolutionary dynamics on phylogenetic trees. *Methods Ecol. Evol.* **5**, 701–707 (2014).

33. Huber, J.T. Biodiversity of Hymenoptera. In: *Insect biodiversity: science and society*. (2017).

34. Pilgrim, E.M., Von Dohlen C.D. & Pitts J.P. Molecular phylogenetics of Vespoidea indicate paraphyly of the superfamily and novel relationships of its component families and subfamilies. *Zool. Scr.* **37**, 539–560 (2008).

35. Branstetter, M.G. et al. Genomes of the Hymenoptera. *Curr. Opin. Insect. Sci.* **25**, 65–75 (2018).

36. Sann, M. et al. Phylogenomic analysis of Apoidea sheds new light on the sister group of bees. *BMC Evol. Biol.* **18**, 71 (2018).

37. Pulawski, W. Catalogue of Sphecidae sensu lato. https://www.calacademy.org/scientists/projects/catalog-of-sphecidae (accessed February, 2021).

38. Buffington, M.L., Forshage M., Liljeblad J., Tang C.-T. & van Noort S. World Cynipoidea (Hymenoptera): A key to higher-level groups. *Insect Syst. Div.* **4**, 1 (2020).

39. Chen, H. et al. An integrated phylogenetic reassessment of the parasitoid superfamily Platygastroidea (Hymenoptera: Proctotrupomorpha) results in a revised familial classification. *Syst. Entomol.* **46**, 1088–1113 (2021).

40. Revell, L. J. Phytools: an R package for phylogenetic comparative biology (and other things). *Methods Ecol. Evol.* **3,** 217–223 (2012).

41. Alfaro, M.E. et al. Nine exceptional radiations plus high turnover explain species diversity in jawed vertebrates. *Proc. Natl. Acad. Sci. U. S. A.* **106**, 13410–13414 (2009).

42. Harmon, L. et al. Package 'geiger'. R package version 2, (2020).

43. Beaulieu, J.M. & O'Meara B.C. Detecting hidden diversification shifts in models of trait-dependent speciation and extinction. *Syst. Biol.* **65**, 583–601 (2016).

44. Rabosky, D.L. & Goldberg E.E. Model inadequacy and mistaken inferences of trait-dependent speciation. *Syst. Biol.* **64**, 340–355 (2015).

45. Uyeda, J.C., Zenil-Ferguson R. & Pennell M.W. Rethinking phylogenetic comparative methods. *Syst. Biol.* **67**, 1091–1109 (2018).

46. O'Meara, B.C. & Beaulieu J.M. Past, future, and present of state-dependent models of diversification. *Am. J. Bot.* **103**, 792–795 (2016).

47. Harrington, S. & Reeder T.W. Rate heterogeneity across Squamata, misleading ancestral state reconstruction, and the importance of proper null model specification. *J. Evol. Biol.* **30**, 313–325 (2017).

48. Vasconcelos, T., O'Meara B.C. & Beaulieu J.M. A flexible method for estimating tip diversification rates across a range of speciation and extinction scenarios. *Evolution* **76**, 1420–1433 (2022).

49. Sharkey, M.J. Phylogeny and classification of Hymenoptera. *Zootaxa* **1668**, 521–548 (2007).

50. Peters, R.S. et al. Evolutionary history of the Hymenoptera. *Curr. Biol.* **27**, 1013–1018 (2017).

51. Davis, R.B., Baldauf S.L. & Mayhew P.J. The origins of species richness in the Hymenoptera: insights from a family-level supertree. *BMC Evol. Biol.* **10**, 109 (2010).

52. Vilhelmsen, L., Miko I. & Krogmann L. Beyond the wasp-waist: structural diversity and phylogenetic significance of the mesosoma in apocritan wasps (Insecta: Hymenoptera). *Zool. J. Linn. Soc.* **159**, 22–194 (2010).

53. Peters, R.S. et al. The taming of an impossible child: a standardized all-in approach to the phylogeny of Hymenoptera using public database sequences. *BMC Biol.* **9**, 1–14 (2011).

54. Heraty, J. et al. Evolution of the hymenopteran megaradiation. *Mol. Phylogen. Evol.* **60**, 73–88 (2011).

55. Sharkey, M.J. et al. Phylogenetic relationships among superfamilies of Hymenoptera. *Cladistics* **28**, 80–112 (2012).

56. Klopfstein, S., Vilhelmsen L., Heraty J.M., Sharkey M. & Ronquist F. The hymenopteran tree of life: evidence from protein-coding genes and objectively aligned ribosomal data. *PLOS One* **8**, e69344 (2013).

57. Sharanowski, B.J. et al. Expressed sequence tags reveal Proctotrupomorpha (minus Chalcidoidea) as sister to Aculeata (Hymenoptera: Insecta). *Mol. Phylogen. Evol.* **57**, 101–112 (2010).

58. Rasnitsyn, A. An outline of evolution of the hymenopterous insects (order Vespida). *Orient. Insects* **22**, 115–145 (1988).

59. Malm, T. & Nyman T. Phylogeny of the symphytan grade of Hymenoptera: new pieces into the old jigsaw (fly) puzzle. *Cladistics* **31**, 1–17 (2015).

60. Vilhelmsen, L. The phylogeny of lower Hymenoptera (Insecta), with a summary of the early evolutionary history of the order. *J. Zoolog. Syst. Evol.* **35**, 49–70 (1997).

61. Vilhelmsen, L. Phylogeny and classification of the extant basal lineages of the Hymenoptera (Insecta). *Zool. J. Linn. Soc.* **131**, 393–442 (2001).

62. Bossert, S., Murray E.A., Blaimer B.B. & Danforth B.N. The impact of GC bias on phylogenetic accuracy using targeted enrichment phylogenomic data. *Mol. Phylogen. Evol.* **111**, 149–157 (2017).

63. Trietsch, C., Miko I., Ulmer J. M., Deans A. R. Translucent cuticle and setiferous patches in Megaspilidae (Hymenoptera, Ceraphronoidea). *J. Hymenopt. Res.* **60**, 135–156 (2017).

64. Ernst, A., Miko I. & Deans A. Morphology and function of the ovipositor mechanism in Ceraphronoidea (Hymenoptera, Apocrita). *J. Hymenopt. Res.* **33**, 25 (2013).

65. Alam, S.M. The skeleto-muscular mechanism of *Sternobracondeesae* Cameron, (Braconidae, Hymenoptera) an Ectoparasite of Sugarcane & Juar Borers of India. Part II. Abdomen and internal anatomy.). Aligarh Muslim University (1953).

66. Eggs, B., Birkhold A.I., Röhrle O. & Betz O. Structure and function of the musculoskeletal ovipositor system of an ichneumonid wasp. *BMC Zoology* **3**, 12 (2018).

67. Sharkey, M.J. & Wahl D.B. Cladistics of the Ichneumonoidea (Hymenoptera). *J. Hymenopt. Res.* **1**, 15–24 (1992).

68. Mikó, I. et al. A new megaspilid wasp from Eocene Baltic amber (Hymenoptera: Ceraphronoidea), with notes on two non-ceraphronoid families: Radiophronidae and Stigmaphronidae. *PeerJ* **6**, e5174 (2018).

69. Ronquist, F. et al. A total-evidence approach to dating with fossils, applied to the early radiation of the Hymenoptera. *Syst. Biol.* **61**, 973–999 (2012).

70. Arcila, D., Alexander Pyron R., Tyler J.C., Ortí G. & Betancur-R R. An evaluation of fossil tip-dating versus node-age calibrations in tetraodontiform fishes (Teleostei: Percomorphaceae). *Mol. Phylogen. Evol.* **82**, 131–145 (2015).

71. Ronquist, F., Lartillot N. & Phillips M.J. Closing the gap between rocks and clocks using total-evidence dating. *Philos. Trans. R. Soc. Lond., B, Biol. Sci.* **371**, 20150136 (2016).

72. Blaimer, B.B. et al. Phylogenomic methods outperform traditional multi-locus approaches in resolving deep evolutionary history: a case study of formicine ants. *BMC Evol. Biol.* **15**, 271 (2015).

73. Drummond, A.J., Suchard M.A., Xie D. & Rambaut A. Bayesian Phylogenetics with BEAUti and the BEAST 1.7. *Mol. Biol. Evol.* **29**, 1969–1973 (2012).

74. Ronquist, F. et al. MrBayes 3.2: efficient Bayesian phylogenetic inference and model choice across a large model space. *Syst. Biol.* **61**, 539–542 (2012).

75. Thomas, G.H. et al. PASTIS: an R package to facilitate phylogenetic assembly with soft taxonomic inferences. *Methods Ecol. Evol.* **4**, 1011–1017 (2013).

76. Chang, J., Rabosky D.L. & Alfaro M.E. Estimating diversification rates on incompletely sampled phylogenies: theoretical concerns and practical solutions. *Syst. Biol.* **69**, 602–611 (2019).

77. Moore, B.R., Höhna S., May M.R., Rannala B. & Huelsenbeck J.P. Critically evaluating the theory and performance of Bayesian analysis of macroevolutionary mixtures. *Proc. Natl. Acad. Sci. U. S. A.* **113**, 9569–9574 (2016).

78. Rabosky, D.L. Phylogenetic tests for evolutionary innovation: the problematic link between key innovations and exceptional diversification. *Philos. Trans. R. Soc. Lond., B, Biol. Sci.* **372**, 20160417 (2017).

79. Kodandaramaiah, U. & Murali G. What affects power to estimate speciation rate shifts? *PeerJ* **6**, e5495 (2018).

80. Meyer, A.L.S. & Wiens J.J. Estimating diversification rates for higher taxa: BAMM can give problematic estimates of rates and rate shifts. *Evolution* **72**, 39–53 (2018).

81. Maddison, W.P., Midford P.E. & Otto S.P. Estimating a binary character's effect on speciation and extinction. *Syst. Biol.* **56**, 701–710 (2007).

82. Uyeda, J.C., Zenil-Ferguson R. & Pennell M.W. Rethinking phylogenetic comparative methods. *Syst. Biol.* **67**, 1091–1109 (2018).

83. Sahoo, R.K., Warren A.D., Collins S.C. & Kodandaramaiah U. Hostplant change and paleoclimatic events explain diversification shifts in skipper butterflies (Family: Hesperiidae). *BMC Evol. Biol.* **17**, 174 (2017).

84. Economo, E.P. et al. Evolution of the latitudinal diversity gradient in the hyperdiverse ant genus Pheidole. *Glob. Ecol. Biogeogr.* **28**, 456–470 (2019).

85. Song, H. et al. Phylogenomic analysis sheds light on the evolutionary pathways towards acoustic communication in Orthoptera. *Nat. Commun.* **11**, 4939 (2020).

86. Blaimer, B.B. et al. Data from: Key innovations and the diversification of Hymenoptera, Dryad, Dataset, https://doi.org/10.5061/dryad.08kprr54m (2023).
