# Supplementary material for: Key innovations and the diversification of Hymenoptera
Source: Nat Commun. 2023 Mar 3;14:1212. doi: 10.1038/s41467-023-36868-4 (PMC9984522; doi:10.1038/s41467-023-36868-4)
Supplement: Supplementary file 3 — Description of Additional Supplementary Files [file 41467_2023_36868_MOESM3_ESM.pdf]

**Description of Additional Supplementary Files**

File Name: Supplementary Information.
Description: Supplementary Methods, Figures, Tables and Discussion referenced in the main text of this article.

File Name: Supplementary Data 1.
Description: Taxon list, voucher deposition and collection information, sequence accessions and results of UCE sequencing.

File Name: Supplementary Data 2.
Description: Specifications for alignments, partitioning and model choice.

File Name: Supplementary Data 3.
Description: GC content per sequence.

File Name: Supplementary Data 4.
Description: Summary statistics for UCE loci.

File Name: Supplementary Data 5.
Description: Results of Four-cluster Likelihood Mapping (FcLM) analyses for major phylogenetic hypotheses.

File Name: Supplementary Data 6.
Description: Summary of divergence age estimations.

File Name: Supplementary Data 7.
Description: Model scores for ancestral character reconstructions of life strategies in Hymenoptera.

File Name: Supplementary Data 8.
Description: Probabilities for ancestral character reconstructions of life strategies in Hymenoptera.

File Name: Supplementary Data 9.
Description: Evolutionary rate dynamics estimated with BAMM for selected clades.

File Name: Supplementary Data 10.
Description: Summary of models tested in the HiSSE framework.

File Name: Supplementary Data 11.
Description: Parameter estimates of the best-fitting models tested in the HiSSE framework.

File Name: Supplementary Data 12.
Description: Tip state probabilities estimated with HiSSE for putative key innovations in Hymenoptera.

File Name: Supplementary Data 13.
Description: Tip rates and states estimated with MiSSE.

File Name: Supplementary Data 14.
Description: FcLM taxon groups.

File Name: Supplementary Data 15.
Description: Information on fossil calibrations.

File Name: Supplementary Data 16.
Description: Comprehensive list for clade specific data used in ancestral character reconstructions, HiSSE and BAMM analyses.

File Name: Supplementary Data 17.
Description: Trait specific sampling frequencies employed in HiSSE.
