# Supplementary material for: Key innovations and the diversification of Hymenoptera
Source: Nat Commun. 2023 Mar 3;14:1212. doi: 10.1038/s41467-023-36868-4 (PMC9984522; doi:10.1038/s41467-023-36868-4)
Supplement: Supplementary file 5 — Reporting Summary [file 41467_2023_36868_MOESM5_ESM.pdf]

nature portfolio

Corresponding author(s): Bonnie B. Blaimer

Last updated by author(s): Feb 8, 2023

Reporting Summary

Nature Portfolio wishes to improve the reproducibility of the work that we publish. This form provides structure for consistency and transparency in reporting. For further information on Nature Portfolio policies, see our Editorial Policies and the Editorial Policy Checklist.

Statistics

For all statistical analyses, confirm that the following items are present in the figure legend, table legend, main text, or Methods section.

n/a	Confirmed	
☒	☐	The exact sample size (*n*) for each experimental group/condition, given as a discrete number and unit of measurement
☒	☐	A statement on whether measurements were taken from distinct samples or whether the same sample was measured repeatedly
☒	☐	The statistical test(s) used AND whether they are one- or two-sided<br>*Only common tests should be described solely by name; describe more complex techniques in the Methods section.*
☒	☐	A description of all covariates tested
☒	☐	A description of any assumptions or corrections, such as tests of normality and adjustment for multiple comparisons
☒	☐	A full description of the statistical parameters including central tendency (e.g. means) or other basic estimates (e.g. regression coefficient) AND variation (e.g. standard deviation) or associated estimates of uncertainty (e.g. confidence intervals)
☒	☐	For null hypothesis testing, the test statistic (e.g. *F*, *t*, *r*) with confidence intervals, effect sizes, degrees of freedom and *P* value noted<br>*Give P values as exact values whenever suitable.*
☒	☐	For Bayesian analysis, information on the choice of priors and Markov chain Monte Carlo settings
☒	☐	For hierarchical and complex designs, identification of the appropriate level for tests and full reporting of outcomes
☒	☐	Estimates of effect sizes (e.g. Cohen's *d*, Pearson's *r*), indicating how they were calculated

Our web collection on statistics for biologists contains articles on many of the points above.

Software and code

Policy information about availability of computer code

Data collection

UCE data were processed using published scripts within the PHYLUCE package (version 1.5.0; Faircloth 2016); assembly of cleaned reads used Trinity (standalone version trinityrnaseq_r20140717). In PHYLUCE v 1.5.0, we used wrapper scripts for the following software: Trimming of demultiplexed FASTQ data output for adapter contamination and low-quality bases used Illumiprocessor v2.0.7, based on Trimmomatic v0.32-1; alignment of sequence data for individual UCE loci used MAFFT v7.130b; internal trimming of misaligned bases used Gblocks v0.91b. UCE sequences for the six non-Hymenoptera outgroup taxa were captured from genome assemblies published on NCBI (see Supplementary Data 1 for accession numbers), using scripts published within the PHYLUCE package (v1.5.0) and outlined in the tutorial "harvesting UCEs from genomes" (https://phyluce.readthedocs.io/en/latest/tutorial-three.html). Extraction of protein-coding loci from captured UCEs followed a custom-pipeline described in Borowiec (2019) with the associated scripts available at https://github.com/marekborowiec/uce-to-protein; some steps with this pipeline rely on BLASTX v2.8.1 (https://blast.ncbi.nlm.nih.gov/Blast.cgi). Data were then aligned with MAFFT v7.130b and internal trimming was performed with Gblocks v0.91b. For further data exploration (see supplementary methods) trimming was performed with TrimALvl.2 and alignment with MUSCLE v3.8.31.

Data analysis	Alignment statistics were calculated with AMASv1.0. The Sliding-Window Site Characteristics Entropy (SWSC-EN) algorithm v1.0.0 (https:// github.com/Tagliacollo/PFinderUCE-SWSC-EN) and Partitionfinder v2.1.1 were used for partitioning of data sets. IQ-TREE v1.6.10 was used for maximum likelihood analysis; IQ-TREE v1.6.12 was used for Four-cluster Likelihood Mapping (FcLM). PAML v4.9j was used for divergence time estimate analysis using MCMCTREE. TracerV1.7.1 was used to assess convergence. For ancestral character state reconstruction, we used the package corHMM v2.5 in R version 4.0.3. For diversification analyses, BAMM v2.5.0, the R package BAMMtools v2.1.7, the R package phytools v0.7-70, and the R package Geiger v2.0.7 were used in R version 4.0.3. Hisse v.1.9.18 was used for trait-dependent diversification analysis in R version 4.0.3. Hisse v2.1.9 was used for MiSSE analyses in R version 4.2.2. No custom code has been used in the analyses. Input files, matrices and tree files are available in the Dryad repository at https://doi.org/10.5061/dryad.08kprr54m.

For manuscripts utilizing custom algorithms or software that are central to the research but not yet described in published literature, software must be made available to editors and reviewers. We strongly encourage code deposition in a community repository (e.g. GitHub). See the Nature Portfolio guidelines for submitting code & software for further information.

Data

Policy information about availability of data

All manuscripts must include a data availability statement. This statement should provide the following information, where applicable:

- Accession codes, unique identifiers, or web links for publicly available datasets
- A description of any restrictions on data availability
- For clinical datasets or third party data, please ensure that the statement adheres to our policy

The raw sequence reads newly generated in this study have been deposited in the NCBI Sequence Read Archive under BioProject accession code PRJNA811764 (https://www.ncbi.nlm.nih.gov/bioproject/PRJNA811764) and PRJNA632862 (https://www.ncbi.nlm.nih.gov/bioproject/PRJNA632862). The UCE sequence data from prior publications used in this study are available under BioProject accession codes PRJNA379583 (https://www.ncbi.nlm.nih.gov/bioproject/PRJNA379583), PRJNA248919 (https://www.ncbi.nlm.nih.gov/bioproject/PRJNA248919), PRJNA495844 (https://www.ncbi.nlm.nih.gov/bioproject/PRJNA495844), PRJNA814466 (https://www.ncbi.nlm.nih.gov/bioproject/PRJNA814466), PRJNA606284 (https://www.ncbi.nlm.nih.gov/bioproject/PRJNA606284), PRJNA647791 (https:// www.ncbi.nlm.nih.gov/bioproject/PRJNA647791), PRJNA625490 (https://www.ncbi.nlm.nih.gov/bioproject/PRJNA625490), and PRJNA473845 (https:// www.ncbi.nlm.nih.gov/bioproject/PRJNA473845); the genome sequence used in this study are available under accession codes GCA_001444175 (https:// www.ncbi.nlm.nih.gov/data-hub/genome/GCA_001444175.2/), GCA_000699045 (https://www.ncbi.nlm.nih.gov/data-hub/genome/GCF_000699045.2/), GCA_000762945 (https://www.ncbi.nlm.nih.gov/data-hub/genome/GCA_000762945.2/), GCA_000696855 (https://www.ncbi.nlm.nih.gov/data-hub/genome/ GCA_000696855.2/), GCA_000006295 (https://www.ncbi.nlm.nih.gov/data-hub/genome/GCF_000006295.1/), and GCA_000931545 (https:// www.ncbi.nlm.nih.gov/data-hub/genome/GCA_000931545.1/). The raw sequence data can also be accessed using individual accession numbers given in Supplementary Data 1. Source data for this study (assembled contig files, sequence data matrices, tree and log files for phylogenetic analyses, input and output files for FcLM, R code and input and results files) are available in the Dryad repository at https://doi.org/10.5061/dryad.08kprr54m. Information on the location of voucher specimens is provided in Supplementary Data 1.

Human research participants

Policy information about studies involving human research participants and Sex and Gender in Research.

Reporting on sex and gender	Not applicable. This study did not involve human research participants.
Population characteristics	Not applicable. This study did not involve human research participants.
Recruitment	Not applicable. This study did not involve human research participants.
Ethics oversight	Not applicable. This study did not involve human research participants.

Note that full information on the approval of the study protocol must also be provided in the manuscript.

Field-specific reporting

Please select the one below that is the best fit for your research. If you are not sure, read the appropriate sections before making your selection.

☐ Life sciences ☐ Behavioural & social sciences ☒ Ecological, evolutionary & environmental sciences

For a reference copy of the document with all sections, see nature.com/documents/nr-reporting-summary-flat.pdf

Ecological, evolutionary & environmental sciences study design

All studies must disclose on these points even when the disclosure is negative.

Study description	This study comprises molecular phylogenetic and macroevolutionary analyses based on ultraconserved element data generated from insect specimens. The taxonomic focus is the insect order Hymenoptera, which includes ants, wasps, bees and sawflies.
Research sample	We assembled a taxon set of 765 species across 94 extant families belonging to 22 superfamilies within the Hymenoptera (ants, wasps, bees and sawflies), and six non-hymenopteran outgroups (Coleoptera (Agrilus planipennis), Diptera (Aedes albopictus), Lepidoptera (Papilio glaucus), Hemiptera (Homalodisca vitripennis), Psocodea (Pediculus humanus corporis), Blattodea (Blattella

germanica). This data of 765 hymenopteran species (i.e. 765 samples) was chosen to assemble a balanced taxon set across the Hymenoptera representing all major lineages. We generated UCE sequence data de novo ourselves for most taxa; sequences for 370 taxa are newly released for publication in the context of this study, while 395 sequences have already been published in other studies by some of us. Data for outgroups were assembled by mining UCEs in silica from published genomes downloaded from NCBI. Specific species representing lineages were chosen based on the availability of DNA-grade tissue samples in museum collections and previously published sequences. Samples represent the entire population of the respective species. Male and female specimens have been sampled, without particular preference. Specimens have collecting year ranges from 1905-2017. Information on taxon sampling with NCBI accession numbers and references is presented in Supplementary Data 1.

Sampling strategy

Our goal was to create a well-sampled, balanced taxon sampling across the order Hymenoptera by combining newly generated sequences with previously published data. Our sampling for newly generated data has an emphasis on the hyperdiverse lineages within the superfamilies Ceraphronoidea, Chalcidoidea, Cynipoidea, Ichneumonoidea and Platygastroidea. The specific taxa chosen to represent lineages were selected based on the availability of DNA-grade tissue samples.

Data collection

RRK, BFS, BBB, MWG, MLB, EJT, IM, DRS, JYR and AC provided insect museum samples, either as pinned dried specimens or preserved in 95-100% Ethanol, or sequence data. No specimens were specifically collected for this study; museum specimens (where information is available) were collected with a variety of methods, including hand collecting/netting, Malaise traps and yellow pan traps. BBB, BFS, JYR and AC carried out DNA extractions, library preparation and target enrichment for UCEs, using protocols summarized in the Supplementary Methods and Data of the article. BBB, BFS and AC assembled and processed UCE data, and BBB performed phylogenetic and macroevolutionary analyses.

Timing and spatial scale

The sequence data for this study were collected between January 2017 and September 2018. This time frame was delimited by the timing of available grant funding for the study. The taxa included in this study stem from museum collections representing global sampling efforts.

Data exclusions

Sequence data were cleaned and trimmed according to standard protocols to remove contamination and sequencing error. After this step, no data were excluded.

Reproducibility

We have provided all data and code used in the study in a Dryad repository. Further, we provided detailed methodology for analyses in the Supplementary Material. We therefore believe that the study is fully reproducible.

Randomization

We performed phylogenetic analyses based on the taxon sampling and molecular sampling outlined, applying models of nucleotide evolution to infer phylogenetic trees using maximum likelihood methods. Phylogenetic methods are fundamentally different from a standard statistical experimental design that requires randomization.

Blinding

The type of questions and phylogenetic analyses carried out in this study do not require blinding experiment design, because there are no participants who may be influenced by the treatments.

Did the study involve field work? ☐ Yes ☒ No

Reporting for specific materials, systems and methods

We require information from authors about some types of materials, experimental systems and methods used in many studies. Here, indicate whether each material, system or method listed is relevant to your study. If you are not sure if a list item applies to your research, read the appropriate section before selecting a response.

Materials & experimental systems

n/a	Involved in the study	
☒	☐	Antibodies
☒	☐	Eukaryotic cell lines
☒	☐	Palaeontology and archaeology
☐	☒	Animals and other organisms
☒	☐	Clinical data
☒	☐	Dual use research of concern

Methods

n/a	Involved in the study	
☒	☐	ChIP-seq
☒	☐	Flow cytometry
☒	☐	MRI-based neuroimaging

Animals and other research organisms

Policy information about studies involving animals; ARRIVE guidelines recommended for reporting animal research, and Sex and Gender in Research

Laboratory animals

This study did not involve laboratory animals.

Wild animals

The insects that were used for generating sequence data in this study were originally collected and killed in the wild, and either directly preserved in 70-100% ethanol for tissue preservation or pinned and dried for morphological study. Since the time of their collection, they have been housed in various museum collections, either preserved in 95-100% Ethanol or stored as dried and pinned specimens. This study did not involve any live animals.

Reporting on sex	Sex was not considered in the study design nor were respective data collected; both male and female specimens have been used indiscriminately in this phylogenomic study. Sex-based analyses are not required for this type of genomic study as no sex-linked loci were studied.
Field-collected samples	No new field collections have been made in the context of this study. The sequence data used in this study came from insect specimens originally collected in the field, but specimens were already accessioned and housed in museum collections.
Ethics oversight	There was no ethical approval or guidance required for this study because the study organisms were common insects not regulated under the IUCN Red List or other ethical committees.

Note that full information on the approval of the study protocol must also be provided in the manuscript.
